# Daily, oral FMT for long-term maintenance therapy in ulcerative colitis: results of a single-center, prospective, randomized pilot study

**DOI:** 10.1186/s12876-021-01856-9

**Published:** 2021-07-08

**Authors:** Jessica W. Crothers, Nathaniel D. Chu, Le Thanh Tu Nguyen, Magen Phillips, Cheryl Collins, Karen Fortner, Roxana Del Rio-Guerra, Brigitte Lavoie, Peter Callas, Mario Velez, Aaron Cohn, Ryan J. Elliott, Wing Fei Wong, Elaine Vo, Rebecca Wilcox, Mark Smith, Zain Kassam, Ralph Budd, Eric J. Alm, Gary M. Mawe, Peter L. Moses

**Affiliations:** 1 Department of Pathology and Laboratory Medicine, University of Vermont Medical Center, 111 Colchester Ave, Burlington, VT 05401 USA; 2 Department of Biological Engineering, Massachusetts Institute of Technology, 77 Massachusetts Avenue, Cambridge, MA 02139 USA; 3 Center for Microbiome Informatics and Therapeutics, Broad Institute, Cambridge, MA USA; 4 Department of Medicine, University of Vermont Medical Center, 111 Colchester Ave, Burlington, VT 05401 USA; 5 Larner College of Medicine, The University of Vermont, 89 Beaumont Ave, Burlington, VT 05401 USA; 6 Flow Cytometry and Cell Sorting Facility, Department of Surgery, Larner College of Medicine, University of Vermont, 89 Beaumont Ave, Burlington, VT 05401 USA; 7 Department of Neurological Sciences, Larner College of Medicine, University of Vermont, 89 Beaumont Ave, Burlington, VT 05401 USA; 8 Department of Medical Biostatistics, University of Vermont, 89 Beaumont Ave, Burlington, VT 05401 USA; 9 OpenBiome, 2067 Massachusetts Ave, Cambridge, MA 02140 USA; 10 Finch Therapeutics, 200 Inner Belt Rd, Somerville, MA 02143 USA

**Keywords:** Fecal microbiota transplantation, FMT, Inflammatory bowel disease, IBD, MAIT cells, Ulcerative colitis, UC, Microbiome, Microbiota

## Abstract

**Background:**

Fecal microbiota transplantation (FMT) is a promising new strategy in the treatment of Inflammatory Bowel Disease, but long-term delivery systems are lacking. This randomized study was designed as a safety and feasibility study of long-term FMT in subjects with mild to moderate UC using frozen, encapsulated oral FMT (cFMT).

**Methods:**

Subjects were randomized 1:1 to receive FMT induction by colonoscopy, followed by 12 weeks of daily oral administration of frozen encapsulated cFMT or sham therpay. Subjects were followed for 36 weeks and longitudenal clinical assessments included multiple subjective and objective markers of disease severity. Ribosomal 16S bacterial sequencing was used to assess donor-induced changes in the gut microbiota. Changes in T regulatory (Treg) and mucosal associated invariant T (MAIT) cell populations were evaluated by flow cytometry as an exploratory endpoint.

**Results:**

Twelve subjects with active UC were randomized: 6 subjects completed the full 12-week course of FMT plus cFMT, and 6 subjects received sham treatment by colonic installation and longitudinal oral placebo capules. Chronic administration of cFMT was found to be safe and well-tolerated but home storage concerns exist. Protocol adherence was high, and none of the study subjects experienced FMT-associated treatment emergent adverse events. Two subjects that received cFMT achieved clinical remission versus none in the placebo group (95% CI = 0.38-infinity, *p* = 0.45). cFMT was associated with sustained donor-induced shifts in fecal microbial composition. Changes in MAIT cell cytokine production were observed in cFMT recipients and correlated with treatment response.

**Conclusion:**

These pilot data suggest that daily encapsulated cFMT may extend the durability of index FMT-induced changes in gut bacterial community structure and that an association between MAIT cell cytokine production and clinical response to FMT may exist in UC populations. Oral frozen encapsulated cFMT is a promising FMT delivery system and may be preferred for longterm treatment strategies in UC and other chronic diseases but further evaluations will have to address home storage concerns. Larger trials should be done to explore the benefits of cFMT and to determine its long-term impacts on the colonic microbiome. Trial registration: ClinicalTrials.gov (NCT02390726). Registered 17 March 2015, https://clinicaltrials.gov/ct2/show/NCT02390726?term=NCT02390726&draw=2&rank=1.

**Supplementary Information:**

The online version contains supplementary material available at 10.1186/s12876-021-01856-9.

## Background

Inflammatory bowel disease (IBD) is a complex chronic inflammatory disease of the gastrointestinal tract, and includes both Crohn’s disease (CD) and ulcerative colitis (UC). While the pathophysiology of IBD remains incompletely understood, these disorders are characterized by immune dysregulation resulting in immune-mediated damage to the alimentary tract. Alterations in the gut microbiota (dysbiosis) have been observed, and are implicated as a key regulatory event in the disease process [1–4]. Most current therapeutic strategies target the immune response directly, but these therapies are associated with high cost and significant risk of adverse events [5, 6]. Modification of the microbial environment by fecal microbiota transplant (FMT) offers an alternative approach that could indirectly influence the host immune system in a safe and less costly manner [7].

FMT is highly efficacious for treating recurrent and refractory *Clostridium difficile* infections, often requiring only a single administration of alternative microbes and resulting in significant and sustained microbial changes in the gut microbiota [8–10]. In four recently published randomized controlled trials that include a total of 277 patients, FMT has demonstrated early clinical promise as a safe, cost effective treatment strategy in a subset of UC patients [11–14]. However, all of these studies included multiple endoscopic or enema-based administrations, raising concerns about the generalizability and long-term feasibility of these approaches.

Recently, oral FMT formulations have shown promise for the treatment of recurrent *C. difficile* infection, demonstrating equivalent clinical efficacy as well as the ability to induce sustained microbial changes in the gut [15–17]. These novel oral cFMT formulations may offer a path forward in the development and widespread applicability of live microbial therapeutics for the treatment of UC and other chronic diseases.

The objective of this preliminary investigation was to investigate the safety and feasibility of using at-home frozen, encapsulated FMT (cFMT) following FMT induction by colonoscopy as a novel, long-term maintenance strategy in the treatment of UC. We also investigated the ability of this novel treatment regimen to induce significant and durable microbial changes utilizing 16S-based compositional analysis, and we assessed if there were longitudinal changes in T cell populations utilizing multicolor flow cytometry.

## Methods

### Study design

This was a single center, double-blinded, placebo-controlled, randomized control trial intended to investigate the safety and feasibility of performing induction FMT by initial colonoscopic infusion followed by 12 weeks of ambulatory oral maintenance therapy with frozen FMT capsules (cFMT). The study protocol was approved by the Institutional Review Board at the University of Vermont (UVM) and the UVM Medical Center (UVMMC) Committee on Human Research in the Medical Sciences (CHRMS) (Additional files 1, 2, 3, 4). All participants provided written informed consent. The study was registered on ClinicalTrials.gov (NCT02390726) under and FDA Investigational New Drug number (IND 16395). After acquisition of all samples, all authors had access to the study data and reviewed and approved the final manuscript.

### Study subjects

Adult subjects were recruited through the IBD Center at UVMMC, or by tertiary or quaternary referral. Study subjects were required to have an established diagnosis of UC, with inflammation extending proximally to at least the recto-sigmoid junction. Subjects with proctitis only were excluded. Subjects were required to be on stable doses of UC-specific medications for at least 6 weeks prior to screening, including anti-TNFα, oral immunomodulators, oral and topical 5-ASA, and methotrexate; cortico-steroid use was excluded. A baseline total Mayo score between 4 and 10, with an endoscopic Mayo subscore ≥ 1, rectal bleeding subscore ≥ 1, and stool frequency subschore ≥ 1, was required for participation. Asymptomatic subjects or those with severe, refractory disease (defined as a Mayo score ≥ 10, or an endoscopic subscore ≥ 3) were excluded, as were patients with a known infectious cause of colitis or exacerbation of baseline symptoms. Patients with a history of colectomy, documented gastrointestinal motility disorder, limited life expectancy (< 12 months), pregnancy, lactation, severe immunodeficiency or a history of anaphylaxis were also excluded. Subjects did not use antibiotics within 6 weeks or probiotics within 4 weeks prior to enrollment. After trial commencement, eligibility criteria were changed to not allow probiotics usage within 1 week prior to enrollment in order to increase recruitment; this change was not expected to undermine the scientific integrity of the study. All study visits and data collection were performed at UVMMC.

### Randomization, blinding and sample size calculation

Eligible subjects were randomized 1:1 by a computer-generated randomization list maintained off-site at OpenBiome (Cambridge, MA) to ensure concealment of allocation and double blinding. The treatment allocation was blinded to the subject, and all on-site investigators and staff. As a pilot feasibility trial, the primary objective of this study was to gain early data on safety and feasibility of longterm cFMT maintenance therapy therefore the target sample size of this study was determined by clinical availability of willing, eligible subjects, and not based on a formal sample-size calculation.

### Baseline assessments

Baseline characterization of participants utilized validated measures of UC activity including the Inflammatory Bowel Disease Questionnaire (IBDQ) and the Mayo symptom score [18, 19]. Endoscopic and histologic disease activity was recorded at week 0 prior to FMT induction and fecal calprotectin levels obtained.

### Donor stool

In order to limit FMT microbial variability and account for any potential FMT donor effect [20], all donor material was derived from two healthy stool donors selected for high (top quintile, 11.11 mmol/g and 12.67 mmol/g stool) butyrate production, as measured by gas chromatography. The short chain fatty acid butyrate has been shown to promote regulatory T cells differentiation and epithelial barrier function [21–24]. In order to mitigate other donor-specific effects, two donors with this phenotype were included in the treatment regimen of all subjects. Donors were rigorously screened by a universal stool bank (OpenBiome, Somerville, MA, USA) and stool allocated to each subject was shipped to the study site frozen on dry ice and maintained at − 20 °C prior to use.

### Antibiotic pretreatment

Subjects in both arms of the study were pretreated with antibiotics (ciprofloxacin 250 mg PO q12 and metronidazole 500 mg PO q8 × 7 days) for 7 days prior to FMT (or placebo) procedure. This regimen was chosen for its ability to disrupt luminal microbial communities prior to FMT and to promote microbiota reprogramming [25].

### Fecal microbiota transplantation induction and maintenance therapy

Each subject in the active treatment arm received fecal material derived from a single donor as induction therapy, delivered by colonoscopic infusion (120 mL at a concentration of 1 g of stool/2.5 mL) following standard bowel preparation. Twelve-week maintenance therapy consisted of an alternating schedule of the same two pre-defined donors at a dose of 1 daily 550μL FMT capsule (~ 0.5 g of stool). Capsules were distributed in 4-week increments, transported on dry ice and maintained in subject’s freezers. Subjects were instructed to follow strict guidelines regarding maintenance of capsule temperature and to not transfer the capsules between freezers, meaning that they could not spend > 24 h away from home during the dosing period. A daily medication adherence log was maintained and monitored at follow-up visits. Subjects allocated to the placebo arm were given sham colonoscopic infusion and sham capsules designed to visually resemble fecal material.

### Clinical outcomes

Clinical follow-up was performed at 4, 8, 12, 18 and 36 weeks with endoscopic and histopathologic evaluations performed at 0 and 12 weeks. Endoscopic and histologic scorings were performed by a single gastroenterologist and surgical pathologist blinded to treatment allocation. Pinch biopsies obtained from the worst affected mucosal surface as determined endoscopically were immediately fixed in 10% formalin and underwent routine tissue processing. Histologic scoring was performed using the Geboes grading system for IBD-associated disease activity [26]. Additional measures of clinical and endoscopic (IBDQ, Mayo and UCEIS scores), as well as inflammatory response (serum CRP, fecal lactoferrin, and fecal calprotectin) were recorded longitudenally. Clinical remission was defined as a modified Mayo Score ≤ 2 at 12 weeks including a rectal bleeding (RB) subscore equal to 0, stool frequency (SF) subscore equal to 0 or with at least a one point decrease from baseline to achieve a SF subscore ≤ 1, and an endoscopic sub-scores of ≤ 1. Clinical response was defined as a decrease in the total Mayo score (SF, RB, physical global assesment, and endoscopic Mayo scores) from baseline of ≥ 3 points with a RB subscore of 0 or 1, or a decrease in the RB subscore of 1 point or more. Adverse events (AEs) were assessed by phone call 24 h following induction, at four clinic visits (weeks 4, 8, 12, and 18) and again by phone call at 36 weeks. AE severity and relatedness were assessed by clinical staff blinded to treatment assignment.

### Stool microbiota analysis by 16S sequencing

Subject stool samples were obtained weekly throughout the study period, beginning prior to antibiotic pretreatment and ending at 18-weeks follow-up. Study subjects were provided with home stool collection kits and instructed to collect samples weekly at roughly the same time of day. Stool collection vials contained RNALater and remained at room temperature during specimen transport. Upon reciept, samples were stored at − 80 °C until processing. DNA extraction was performed using the MoBio Powersoil 96 kit with minor modifications and 16S rRNA gene libraries targeting the V4 region of the 16S rRNA gene were prepared. Each sample was given a unique reverse barcode and replicates were then pooled, cleaned and normalized prior to sequencing on an Illumina MiSeq 300. Raw sequence reads were then processed and OTU calling performed using the Qiime2—dada2 pipeline. Measures of microbial alpha diversity (Shannon index) and beta diversity (Jensen-Shannon divergence) between subjects and donor samples, and to their own baseline samples, were calculated.

### Immunologic profiling of peripheral blood T cells

Dynamic evaluation of lymphocyte subpopulations and cytokine production was performed in subjects at baseline and at weeks 4, 8, and 12 during the maintenance period. Control peripheral blood from healthy individuals without gastrointestinal disease and/or immunodeficiency (n = 10) were obtained at a single time point. Peripheral blood mononuclear cells (PBMC) were isolated via Ficoll gradient centrifugation, and cell staining protocols optimized to assess TCRαβ (CD4+ and CD8+) subsets. Special attention was paid to mucosal associated invariant T cells (MAIT), defined herein as TCR αβ+ CD4+ MR1+, and T regulatory cells (TCRαβ+ CD4+ CD25^hi^). Intracellular cytokine production (IFNγ, IL-10 and IL-17A) was measured by flow cytometry following 5 h of ex vivo stimulation with phorbol myristate acetate and ionomycin. Details of cell processing and staining are provided in the supplementary material. Immunophenotyping was performed with a flow cytometer FACS LSRII (BD Biosciences, San Jose, CA) using fluorochrome-labeled monoclonal antibodies (TCR αβ (AF 488), CD45 (AF 700), CD8 (BUV395), CD4 (BV510), CD13 (PE/Dazzle), CD25 (BV650), hMR1 (NIH tetramer facility APC, 5-OP-RU 2017-04-07, Atlanta, GA), IL-17A (BPerCP/Cy5.5), IL-10 (PE/Cy7), IFN-γ (PE). Data were analyzed with FlowJo software (v 10.4.1, Tree Star, Inc., Ashland, OR).

### Statistical analysis

Relative risks were computed for clinical endpoints (remission and response). Due to the small sample size, exact 95% confidence intervals were obtained using a score statistic and Fisher’s exact test was used to determine *p* values for each comparison. Adverse events were compared using a modified intention to treat analysis to include all subjects receiving at least one study treatment. Differences, including in AE frequency, were compared by Student’s *t*-test or Fisher’s exact test. A mixed model analysis was applied to longitudinal values and paired *t* tests used for within-group comparisons of continuous variables and McNemar’s chi square test was used for categorical variables. For descriptive statistics, means and standard deviations were computed for continuous variables and proportions were computed for categorical variables. Between-group comparisons were conducted using two-sample *t* tests for continuous variables and Fisher’s exact test for categorical variables. These analyses were conducted using SAS version 9.4 (Cary, NC: SAS Institute Inc.) and Prism software (version 7.0a; GraphPad Software, San Diego, CA). Differences with *p* values ≤ 0.05 were considered significant.

## Results

### Patient characteristics

**Fig. 1:**
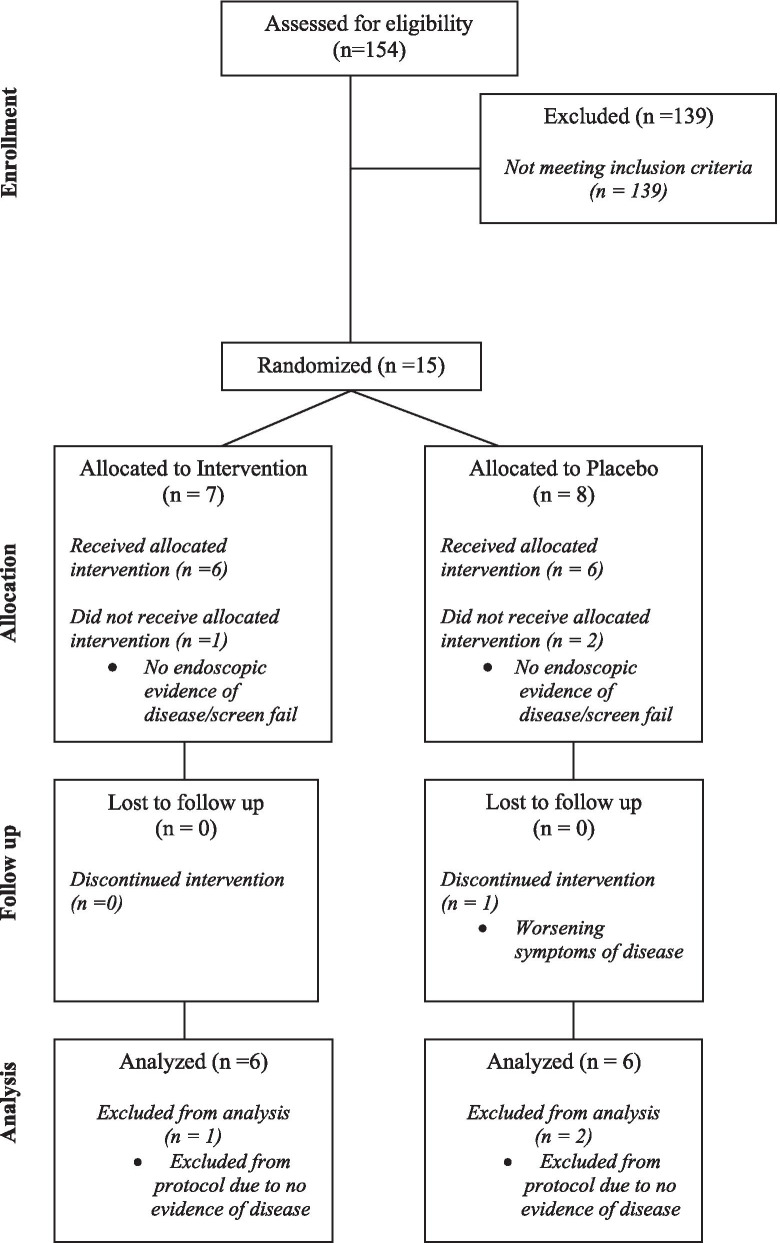
CONSORT diagram showing the flow of subjects through the study. Following randomization, but prior to administration of designated intervention, 1 subject in the treatment group and 2 subjects in the placebo group had no evidence of disease upon endoscopic evaluation and were excluded from the remainder of the study

From February 2016 to September 2017, 154 UC patients were assessed for eligibility, and ultimately 15 subjects were recruited and randomized. Desipite an initial target enrollment of 20 subjects, enrollment was terminated early due to difficulties in recruiting local patients who met all inclusion and exclusion criteria. Of the 15 recruited subjects, 7 individuals were randomly assigned to the FMT and 8 to the placebo arm. Three subjects (1 in the FMT and 2 in the placebo group) did not meet endoscopic criteria for inclusion (Mayo score ≥ 1) and were excluded from the study (Fig. 1). The remaining 12 subjects (6 in each group) received at least one dose of study treatment. While all 6 subjects allocated to the FMT arm completed all treatments and follow-up assessments, 1 patient in the placebo group dropped out at 6 weeks due to worsening disease. The two study groups exhibited comparable baseline demographic and clinical characteristics (Table 1).

**Table 1:** Baseline subject demographics

Variable		Group	
		Active	Placebo
N		6	6
Age	Mean (SD)	41 (15)	52 (15)
Sex	#(%) Male	4 (67%)	3 (50%)
Duration UC	Mean (SD) yrs	8.9 (9.1)	9.8 (10.6)
BMI*	Mean (SD)	24 (3)	29 (4)
Treatment with biologic	#(%) yes	2 (33%)	1 (17%)
Fecal calprotectin	Mean (SD)	573 (659)	408 (277)
Total Mayo score	Mean (SD)	6.3 (2.0)	6.7 (1.2)
Mayo symptom subscore	Mean (SD)	4.8 (1.5)	4.3 (1.0)
Mayo endoscopic subscore	Mean (SD)	1.5 (0.8)	2.3 (0.5)
Endoscopic UCEIS^ score	Mean (SD)	7.0 (1.8)	8.5 (1.8)
Histologic Severity Score**	Mean (SD)	3.4 (1.2)	4.3 (2.0)
IBDQ^^ total score	Mean (SD)	142.8 (16.8)	120.2 (25.1)
IBDQ bowel system subscore	Mean (SD)	4.2 (0.7)	4.3 (0.9)

Baseline clinical characteristics of subjects randomized to both the active FMT and placebo study arms

*Body Mass Index

**Geboes Score

^Ulcerative Colitis Endoscopic Index of Severity

^^Inflammatory Bowel Questionnaire

### Safety evaluation

Among study subjects that received at least one dose of active or placebo therapy, adverse events possibly or probably related to FMT were few (4 total) and were equally distributed between groups (2/6 vs 2/6; *p* = 1.00) (Table 2). The only serious adverse event was a worsening of disease activity, which occurred in one subject from each group. Both of these subjects required escalation of therapy (prednisone taper) during the treatment period (at 6 and 4 weeks following initial FMT, respectively). Mild adverse events included nausea (36 h prior to colonoscopic delivery of placebo material) and fever (24 h following FMT). Notably, this febrile patient also reported fever 24 h prior to the initial FMT procedure, making causality uncertain. No infectious complications occurred.

**Table 2:** Adverse events by treatment assignment

Adverse events	FMT (n = 6)	Placebo (n = 6)	*p* value
AE possibly or probably related to FMT or sham FMT, n (%)	2/6 (33)	2/6 (33)	1.0

AE type and severity, n (%)			
Nausea, mild	0	1 (50)	1.0
Fever, mild	1 (50)	0	1.0
Worsening disease, severe	1 (50)	1 (50)	1.0

Adverse events by treatment group that were possibly or probably related to FMT

Comparisons were made by Fisher’s exact test

Frozen FMT capsules were distributed to subjects in 4-week allotments (28 pills). cFMT capsules were maintained in home freezers for a total of 84 doses per subject. Medication adherence logs revealed strong adherence with < 1% of missed doses across both arms (9/1008); however, many subjects expressed frustration regarding the strict study guidelines imposed to ensure capsule temperature stability, particularly travel restrictions. No subjects undertook over-night travel during the 12-week dosing period.

### Clinical and histologic outcomes

**Fig. 2:**
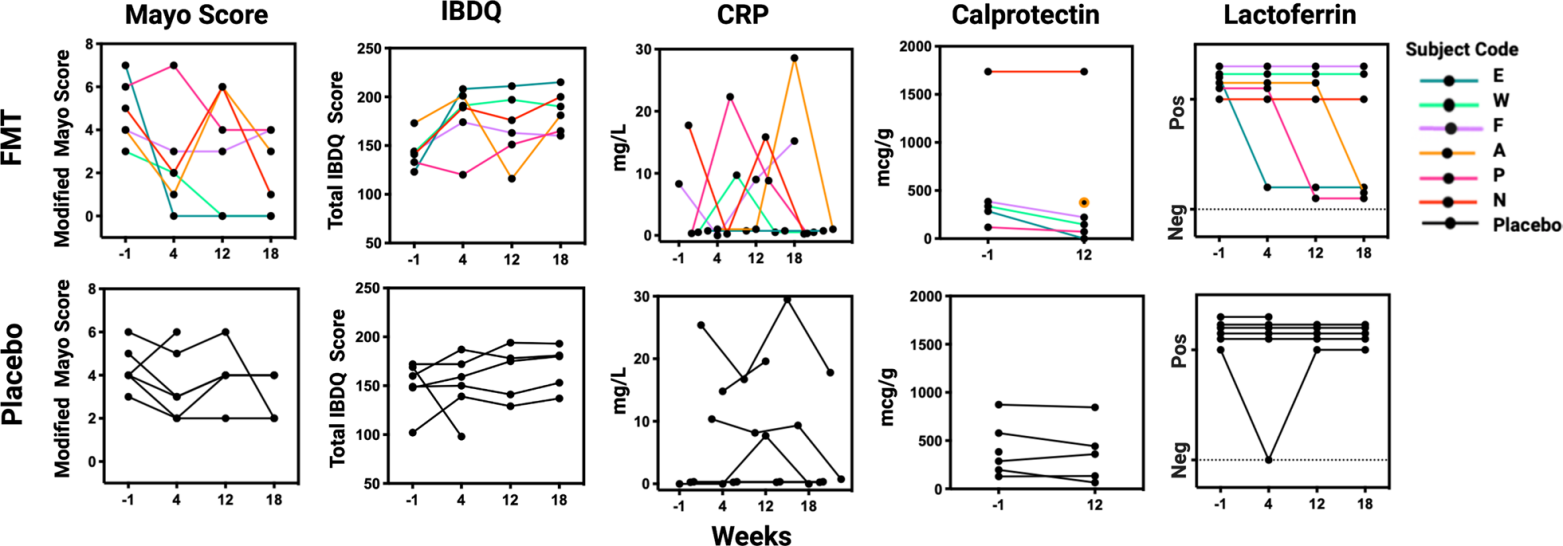
Longitudenal markers of clinical disease and inflammation. Each line represents a single subject over time. Modified Mayo Score includes subject-reported rectal bleeding, stool frequency, and physician global assessment. IBDQ, Inflammatory Bowel Disease Questionairre; CRP, serum C-Reactive Protein (mg/L); Calprotectin (mcg/g) and Lactoferrin (positive/negative) were measured in stool

**Fig. 3:**
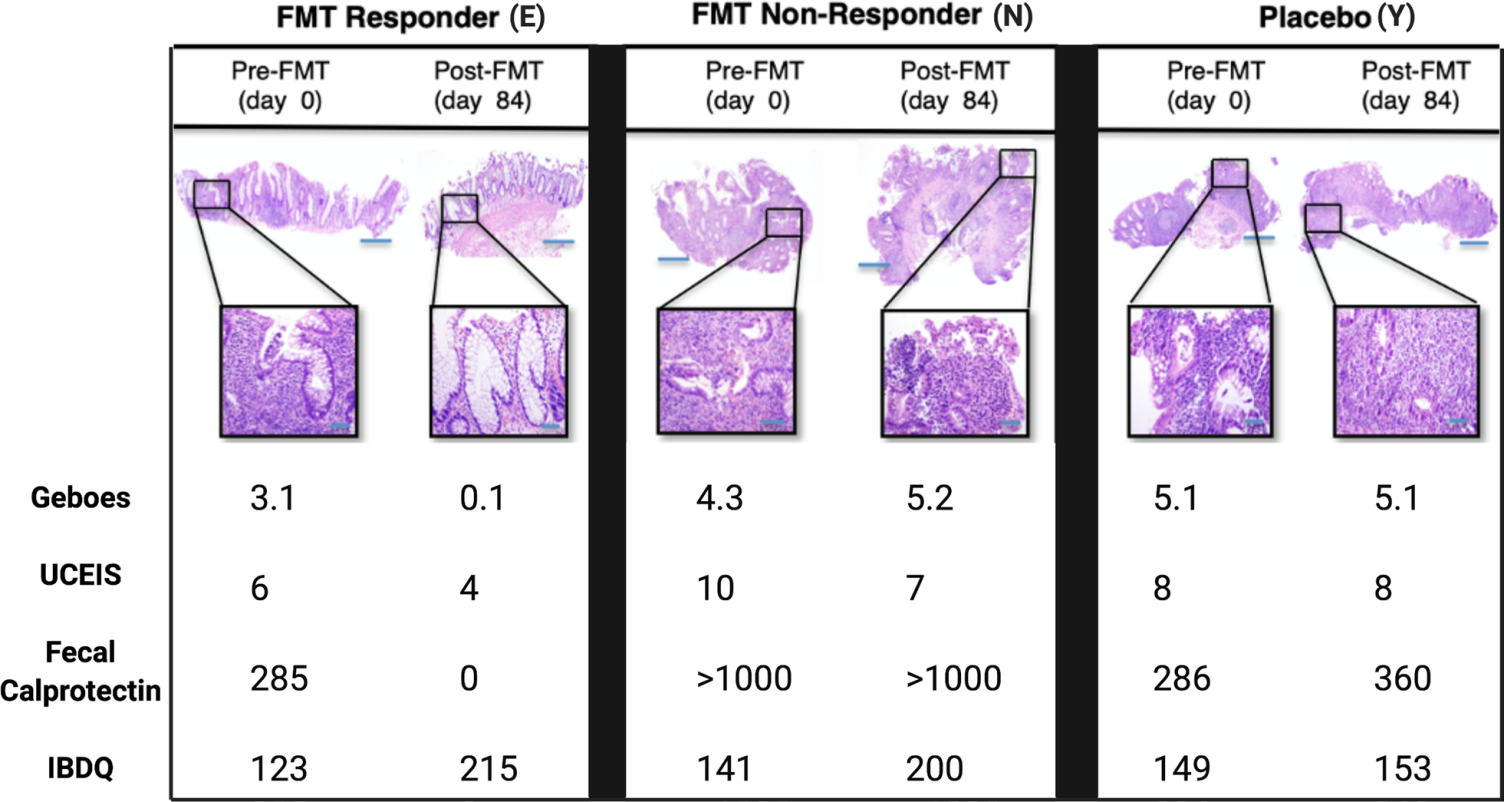
Histologic, endoscopic and clinical parameters of a representative FMT responder (E), non-responder (N), and placebo subject (Y) before and after treatment. Hematoxylin–eosin staining of intestinal mucosa highlight acute and chronic changes and are accompanied by Geboes score (0, structural change only; 1, chronic inflammation; 2, lamina propria neutrophils; 3, neutrophils in epithelium; 4, crypt destruction; and 5, erosions or ulcers), 2x, insets at 20x, scale bar, 50 µm; UCEIS, Ulcerative Colitis Endoscopic Index of Severity; fecal calprotectin (mcg/g), and IBDQ, inflammatory bowel disease questionnaire (scale ranging from 32 (worst) to 224 (best))

At baseline, no significant differences in histologic or endoscopic scoring were detected between the two groups (Table 1). Per subject longitudenal clinical outcome data is provided in Fig. 2. At 12-week follow-up, the mean endoscopic UCEIS score decreased from 7.0 ± 1.8 to 6.2 ± 2.3 in the FMT group and from 8.0 (1.4) to 7.6 (1.8) in the placebo group (*p* = 0.60). The mean histologic Geboes score decreased from 3.4 ± 1.2 to 2.3 ± 2.4 in FMT-treated subjects, and from 4.0 ± 2.1 to 3.8 ± 2.0 in the placebo group (*p* = 0.28). Fecal calprotectin levels decreased from 573 ± 659 to 298 ± 428 in FMT-treated subjects, and from 413 ± 309 to 369 ± 309 in the placebo group. The difference in change of fecal calprotectin between the two groups approached statistic significance (*p* = 0.08). Alternatively, mean CRP levels increased in both groups, from 4.3 ± 7.3 to 10.12 ± 10.43 in the FMT group and from 8.1 ± 10.1 to 10.65 ± 11.31 in the placebo group with no difference in the proportion of subjects with levels > 0 mcg/g noted between groups (*p* = 1.0). While this study was not powered to predict a clinical response, per subject and per groupvalues for markers of clinical and physiologic disease activity are presented for informative purposes (Tables 3, 4). In total, two of six (2/6) subjects (subjects E, and W) in the FMT group achieved clinical remission versus none (0/6) in the placebo group (RR = infinity; CI: 0.38-infinity; *p* = 0.45) and three of six (3/6) subjects (E, W, P) in the FMT group met the study definition of clinical response versus one (1/6) in the placebo group (subject B) (RR = 3.00; CI: 0.42–21.20, *p* = 0.55). It is worth noting that the FMT subject (subject P) who met the definition of clinical response but not remission, required steroid therapy in the middle of the intervention period (week 6), making any attributions of clinical improvement to FMT difficult. FMT subjects achieving clinical remission (E, W) were considered “FMT responders” for the purposes of additional exploratory analyses, including both immunologic and microbiota-based investigations. Representative photomicrographs of biopsy samples from an FMT-responder and non-responders obtained before and after treatment are shown in Fig. 3.

**Table 3:** Changes in clinical, endoscopic, and histologic evidence of disease by subject

	Study code	Age	Sex	Extent of disease	Duration of disease (yrs)	Maintenance therapy	BMI	Change in total Mayo score	Change in endoscopic UCEIS score	Change in endoscopic Mayo score	Change in histologic score	Change in fecal calprotectin (mcg/g)	Change in total IBDQ score
FMT	E	46	F	Pan-colitis	5.5	Mesalamine	20.9	− 7	− 2	0	− 3	− 285	92
	W	35	M	Pan-colitis	7.5	Vedolizumab	27.8	− 3	− 1	0	− 1.3	− 189	47
	F	20	M	Pan-colitis	3.8	Mesalamine	25	− 1	− 1	0	− 2	− 164	16
	A	65	F	L-sided	26.2	Mesalamine	20.9	3	0	1	− 1.2	? = > 375	8
	N	44	M	Pan-colitis	0.2	Sulfasalazine	25.6	1	− 3	0	0.9	> 1000 = > > 1000	59
	P	38	M	Pan-colitis	10.2	Mercaptopurine	25.2	− 3	2	− 1	0	− 42	32
Placebo	B	68	M	Pan-colitis	4.4	Mesalamine	28.8	− 2	− 1	0	0.8	− 132	21
	G	58	M	L-sided	27.8	Mesalamine	26.9	1	0	0	− 2	4	32
	Y	65	M	L-sided	0.4	Mesalamine	36.15	0	0	0	0	74	4
	V	47	F	Pan-colitis	8.8	Adalimumab	29.2	− 1	0	0	0	27	35
	T	31	F	Pan-colitis	0.8	Mesalamine	29.1	0	− 1	0	0	− 137	32
	I	40	F	Pan-colitis	16.3	Mesalamine	25	Dropped out due to worsening disease activity					

IBDQ, Inflammatory Bowel Disease Questionnaire; L-Sides, left-sided disease; BMI, Body Mass Index; yrs, years; wks, weeks

^Ulcerative Colitis Endoscopic Index of Severity

*Geboes Score

**Table 4:** Changes in clinical, endoscopic, and histologic evidence of disease by group

Variable	Group		Screen or procedure	12 week	[12 wk]–[Bl]	*P* value*
CRP	Active	#(%) > 0	2 (33%)	4 (67%)	2 (33%)	0.16
	Placebo	#(%) > 0	2 (40%)	3 (60%)	1 (20%)	0.32
	Active–Placebo					1.00
Fecal calprotectin	Active	Mean (SD)	573 (659)	298 (428)	− 275 (246)	0.07
	Placebo	Mean (SD)	413 (309)	369 (309)	− 44 (90)	0.34
	Active–Placebo				− 231 (185)	0.08
Fecal lactoferrin	Active	#(%) positive	7 (100%)	4 (67%)	0 (0%)	–
	Placebo	#(%) positive	6 (75%)	5 (100%)	0 (0%)	–
	Active–Placebo					–
Endoscopic UCEIS score	Active	Mean (SD)	7.0 (1.8)	6.2 (2.3)	− 0.8 (1.7)	0.29
	Placebo	Mean (SD)	8.0 (1.4)	7.6 (1.8)	− 0.4 (0.5)	0.18
	Active–Placebo				− 0.4 (1.3)	0.60
Endoscopic Mayo score	Active	Mean (SD)	1.5 (0.8)	1.5 (0.5)	0 (0.6)	1.00
	Placebo	Mean (SD)	2.2 (0.4)	2.2 (0.4)	0 (0)	–
	Active–Placebo				0 (0.5)	1.00
Mayo symptom score	Active	Mean (SD)	5.0 (1.5)	3.5 (3.2)	− 1.5 (3.4)	0.33
	Placebo	Mean (SD)	4.2 (1.1)	4.0 (1.4)	− 0.2 (1.1)	0.70
	Active–Placebo				− 1.3 (2.6)	0.44
Histology (Geboes Score)	Active	Mean (SD)	3.4 (1.2)	2.3 (2.2)	− 1.1 (1.4)	0.11
	Placebo	Mean (SD)	4.0 (2.1)	3.8 (2.0)	− 0.2 (1.0)	0.63
	Active–Placebo				− 0.9 (1.2)	0.28
IBDQ bowel system	Active	Mean (SD)	4.2 (0.7)	5.2 (1.4)	1.0 (1.6)	0.19
	Placebo	Mean (SD)	4.1 (0.8)	4.8 (1.2)	0.6 (0.7)	0.11
	Active–Placebo				0.3 (1.3)	0.67
IBDQ emotional health	Active	Mean (SD)	4.4 (0.9)	5.3 (0.8)	0.9 (1.6)	0.23
	Placebo	Mean (SD)	4.9 (1.1)	5.3 (1.0)	0.4 (0.4)	0.09
	Active–Placebo				0.5 (1.2)	0.53
IBDQ systemic systems	Active	Mean (SD)	4.4 (1.2)	4.9 (1.0)	0.5 (2.0)	0.53
	Placebo	Mean (SD)	4.2 (1.1)	4.6 (0.9)	0.5 (0.9)	0.31
	Active–Placebo				0.0 (1.6)	0.96
IBDQ social function	Active	Mean (SD)	5.0 (0.5)	5.7 (1.6)	0.6 (1.3)	0.27
	Placebo	Mean (SD)	5.1 (1.2)	5.9 (1.0)	0.8 (0.5)	0.02
	Active–Placebo				− 0.2 (1.0)	0.79
IBDQ total score	Active	Mean (SD)	142.8 (16.8)	169.0 (34.0)	26.2 (48.4)	0.24
	Placebo	Mean (SD)	146.4 (26.1)	163.4 (27.2)	17.0 (14.4)	0.06
	Active–Placebo				9.2 (37.3)	0.69

Calprotectin (mcg/g) and Lactoferrin (pos/neg) were measured in stool

CRP, serum C-reactive Protein (mg/L); Endoscopic UCEIS, Ulcerative Colitis Endoscopic index of Severy; Mayo symptom score includes subject-reported rectal bleeding, stool frequency, and physician global assessment, IBDQ, Inflammatory Bowel Disease Questionairre

*For within-group comparisons, paired *t* tests were used for continuous variables and McNemar’s chi square test was used for categorical variables. Between-group comparisons were conducted using two-sample *t* tests for continuous variables and Fisher’s exact test for categorical variables

Placebo subjects exhibited inconsistent changes over time, with no clear improvements in symptomatology or clinical evidence of disease. Four of six (66%) placebo subjects required escalation or adjustments in their pharmacologic treatment regimens: one (subject I) at week 4 (this subject dropped out of the trial at this point) and the other 3 study subjects at the end of the treatment period (weeks 12 and 13).

### Longitudinal phenotyping of peripheral blood T-cells

**Fig. 4:**
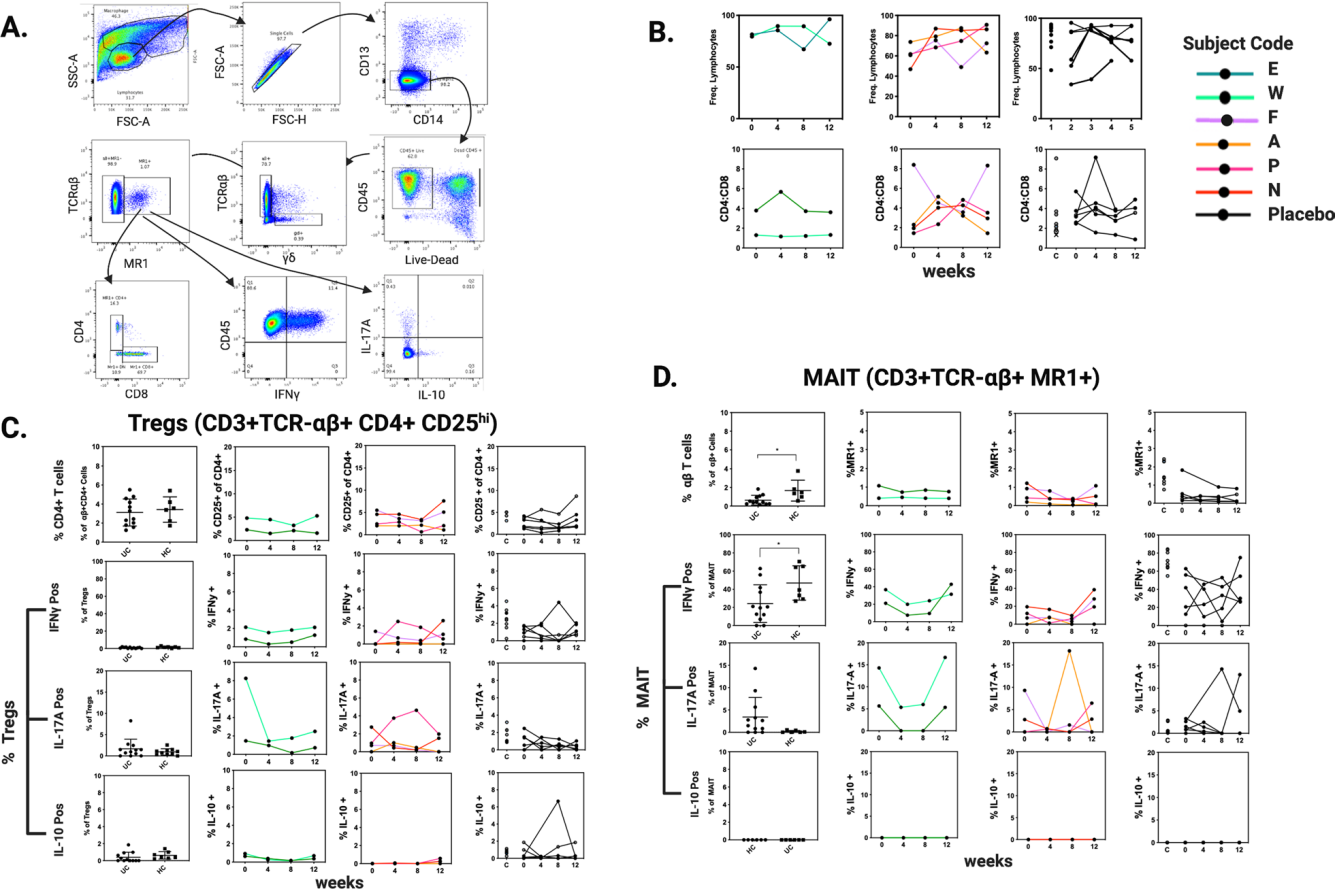
Longitudinal T cell profiling of subjects by flow cytometry and intracellular cytokine staining. **a** Representative gating scheme showing MAIT cell identification and their cytokine production; **b** Frequency of total lymphocytes within peripheral blood mononuclear cell isolations and their CD4:CD8 T cell ratios. **c**, **d** Comparison of T regulatory and MAIT cell frequencies between UC patients and healthy controls (HC) with longitudinal frequencies and INFγ+, IL-17A+, and IL-10+ proportions displayed by treatment group and clinical response (black, placebo; red, non-responser; green, responser). Each line is an individual subject. Controls are displayed with placebo results (C). Between-group comparisons were conducted using two-sample *t* tests and *p* values < 0.05 considered significant

Baseline T cell populations of interest were first compared between UC subjects and healthy controls. The frequency of total lymphocytes obtained following PBMC separation, as well as the CD4:CD8 ratio, were similar between groups. T regulatory cell frequencies were also similar (mean of 3.12% ± 0.41 in UC patients vs. a mean of 3.42% ± 0.54 in controls) with comparable proportions positive for IL-17A and IL-10. No T regulatory cells were IFNγ+. The frequency of mucosal-associated invariant T (MAIT) cells was decreased in UC patients (0.62% ± 0.15 vs. 1.67% ± 0.46) and IL-17A positivity occurred almost exclusively in UC-derived MAIT cell populations (3.42 ± 1.27 vs. 0.1759 ± 0.09). Alternatively, IFNγ secretion was increased in MAIT cells from healthy controls (46.97 ± 7.15 vs. 24.16 ± 6.01), (Fig. 4).

T cell populations were examined before FMT, and then at weeks 4, 8, and 12 during the cFMT maintenance period. By week 4, the frequency of total PBMC lymphocytes and CD4:CD8 T cell ratios increased in the majority of FMT non-responders, while variable changes were observed placebo subjects. One notable exception was subject F who showed a prominent decrease in CD4:CD8 ratio through week 8, after which a reverse dynamic to baseline occurred. Longitudinal frequencies of T regulatory and MAIT cell populations remained relatively constant across groups. By week 4, IL-17A + MAIT and IFNγ + MAIT cells decreased in FMT responders, remained suppressed through week 8, and then returned to baseline by week 12. The number of subjects is insufficient to evaluate the statistical significance of these observed changes.

### Intestinal microbiota analysis by 16S sequencing

#### Relative abundances

**Fig. 5:**
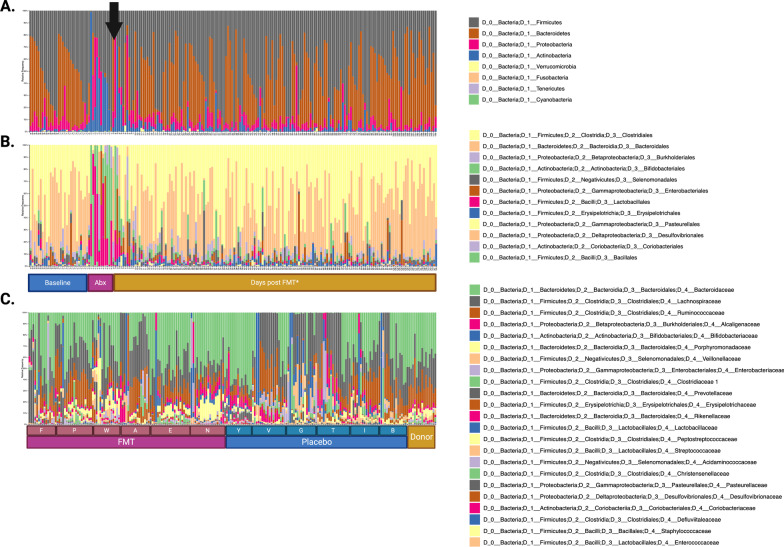
Relative abundance of fecal microbiota in subjects with ulcerative colitis (UC) before and after treatment at the phylum and genus levels. Different colors represent different bacterial species, each bar represents one patient sample. **a**, **b**. most abundant taxa by phylum and genus level, respectively. Arrow denotes day of FMT (*or placebo); **c** 23 most abundant taxa of donors and subject at species level, arranged by subject, treatment group, and day

**Fig. 6:**
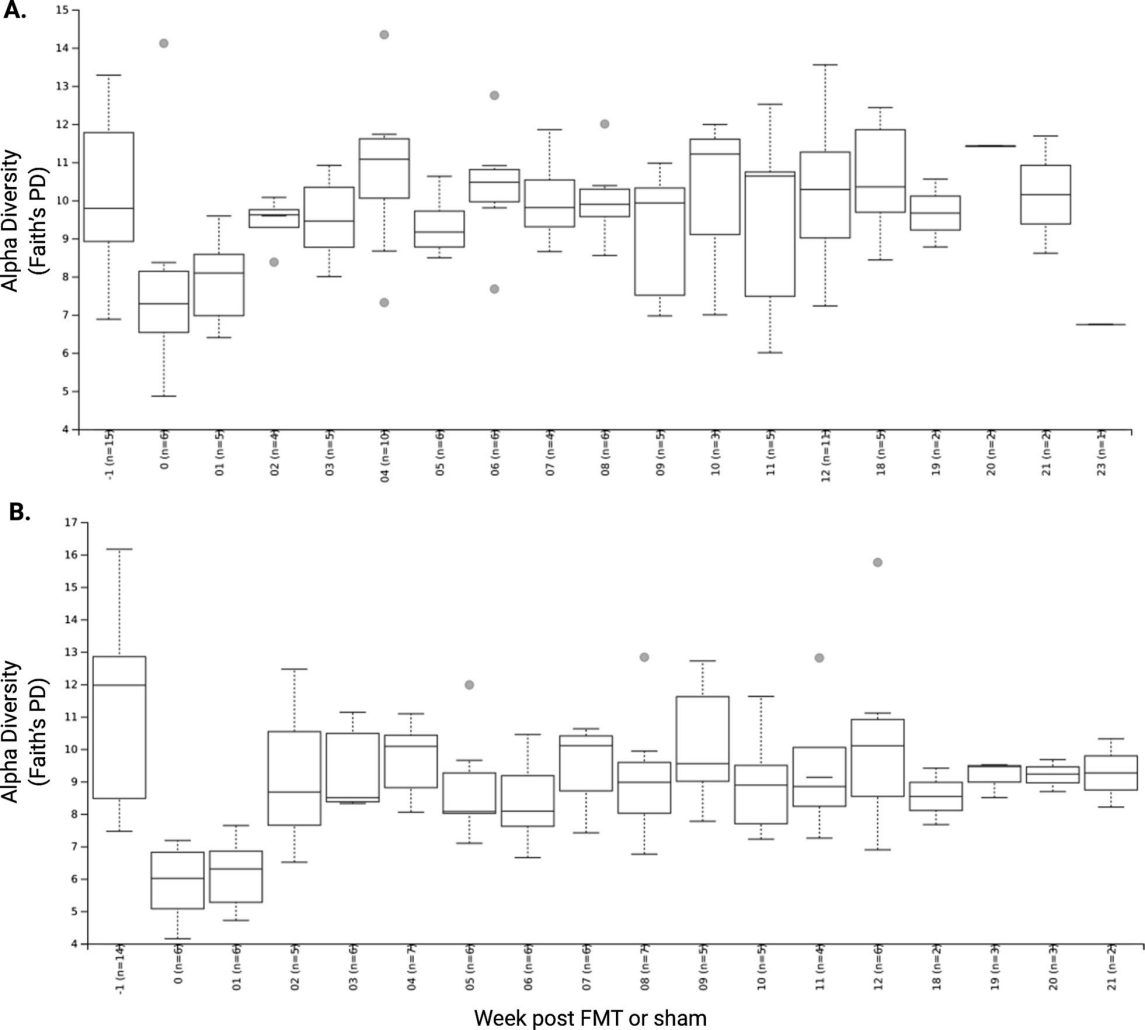
Alpha diversity measured by Shannon index. **a** alpha diversity in placebo subjects grouped by week; **b** alpha diversity in FMT subjects grouped by week. Comparisons between groups made by Student’s *t*-test and *p* values of < 0.05 were considered significant

**Fig. 7:**
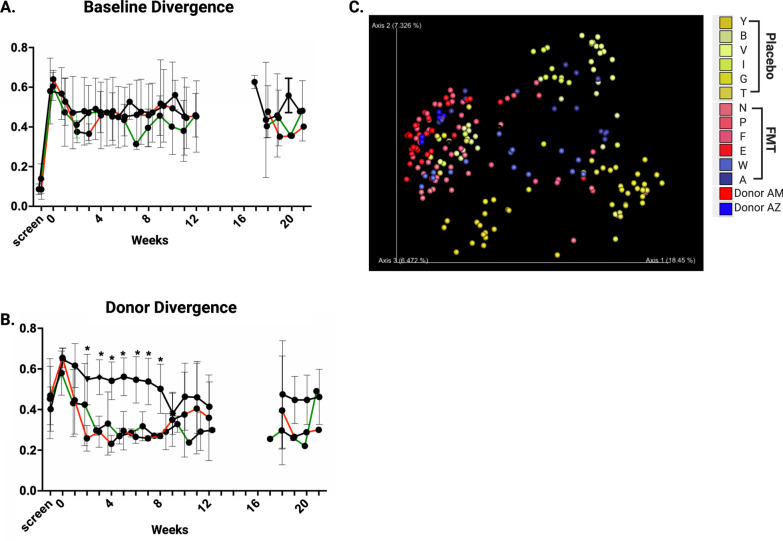
Beta diversity measured by Jenson-Shannon diversity. **a** Beta diversity comparing subjects to their own baseline overtime; **b** Beta diversity comparing subjects to donors. Aggregate data is presented by treatment and clinical response (black, placebo; red, non-responder; green, responder). Comparisons between groups made by Student’s *t*-test and *p* values of < 0.05 were considered significant; **c** Principle component analysis of donors and study subjects. Each dot represents one sample, and subjects are colored by the treatment group (yellow shades represent placebo, blue and red represent primary donor)

Across all time points, stool samples were dominated at the phylum level by *Firmicutes,* and *Bacteroidetes*, which accounted for 88.90% of all sequence reads. Bacteria present at lower proportions included *Proteobacteria,* and *Actinobacteria*, accounting for 6.9% and 4.0% of total reads, respectively. At the genus level, samples were dominated by *Clostridiales* and *Bacteroidales,* with a lower proportion of *Burkholderiales, Bifidobacteriales, Selenomonadlaes, Enterobacteriales, Lactobacillales* observed at various time points. A strong antibiotic effect was observed following a 7-day course of Metronidazole and Ciprofloxacin in all subjects. Changes included a decrease in gram negative and anaerobic bacteria of the *Firmicutes* and *Bacteroidetes* phyla and an increase in gram positive *Actinobacteria,* including from the genus *Bifidobacteriales,* and *Lactobaccilales* Fig. 5. This effect was associated with a decrease in alpha (Shannon) diversity and increased divergence (Jensen-Shannon) from baseline. While these changes were mitigated by the cessation of antimicrobials, neither group returned to their own baseline by 18-week follow-up (Figs. 6, 7).

### Alpha and beta diversity

No difference in alpha or beta diversity was observed between treatment groups at baseline (Fig. 6). FMT did not increase alpha (Shannon) diversity in recipients but did lead to community-level changes in the gut microbiota creating measurable similarity (beta diversity, Jensen-Shannon divergence index) between FMT subjects and their donor. This convergence, which we termed ‘Donor Divergence Index’, remained statistically significant through 8 weeks of dosing (p < 0.01) and although losing significance (*p* = 0.16), could still be detected at week 20, > 8 weeks following cessation of oral cFMT therapy (Fig. 7).

## Discussion

FMT is a promising new therapy that may alleviate the microbial dysbiosis observed in IBD. Practical dosing strategies, however, are needed to test and opptimize the clinical efficacy of long term FMT-based treatment strategies. The goal of the current study was to evaluate the safety and subject tolerance of a treatment plan that involved colonoscopic FMT followed by maintenance treatments of daily cFMT. In this small prospective, randomized controlled trial, we found daily oral FMT to be extremely well tolerated with stronge adherence to the treatment plan. Only very minimal side effects that could be attributed to the treatment were detected, and importantly, no infectious complications occured. Only one other study is known to us in which daily cFMT was trialed in the treatment of UC [27]. In it, 7 subjects took 25 frozen FMT capsules daily (~ 12 g of fecal matter) for 50 days in an open label trial design. As the authors did not report on adverse events, this is the first study to our knowledge to provide early evidence regarding the safety and tolerability of cFMT in UC patients and despite the small sample size, we feel that this study adds to the collective knowledge base of FMT and provides early evidence that oral capsule FMT formulations are safe and without significant side effects in this patient population.

Of note, the logistical challenges presented by frozen oral FMT capsules were considerable. Concerns regarding freeze–thaw cycles incurred by patient transport, home freezer conditions, and potential travel necessitated the use of dry ice during transport, and strict travel guidelines (no overnight travel). While our study population managed to adhere to study guidelines, recording < 1% missed doses during the 12-week study period, we believe that these issues are considerable enough to challenge the practicality of real-world clinical use of frozen FMT capsules for control of UC and other chronic diseases. Moreover, temperature stable formulations should be pursued. Lyophilized fecal preparations or derived bacterial communities may offer viable solutions [28].

While this study was not adequately powered to evaluate the effects of treatment on clinical outcome, we provide additional data to assess the potential impact of FMT on UC disease severity, including clinical, endoscopic, and histologic markers of disease. Of all the clinical markers of disease included in this study, we found fecal calprotectin levels to correlate well with a subject’s clinical course while serum CRP levels were inconsistent. Additionally, our results suggest a divergent clinical response to FMT may exist in which patients separate into “responder” and “non-responder” phenotypes. While varying clinical responses are difficult to interpret in the context of a relapsing and remitting disease like UC, this observation is consistent with previous reports suggesting that FMT efficacy may be limited to a subset of UC patients [29]. What defines patient subsets poised to respond favorably or not to FMT is of significant importance. While our data are too small to assess differences in baseline characterics between responders and non-responders, we noted that non-responders tended to have more severe mucosal damange at baseline as noted by increased histologic and endoscopic scores. The immunomodulating effects of the gut microbiota are well-established [30, 31] and, in the setting of increased gross and microscopic mucosal ulceration, FMT may have the capacity to induce uncontrolled and potentially damaging mucosal inflammation secondary to bacterial translocation and systemic immune activation [32, 33]. Targeting of UC patients with mild to moderate, as opposed to severe disease may therefore be advantageous. Future studies addressing this question are of great interest.

Longitudinal phenotyping of peripheral T-cell populations provides additional insights into the differential clinical responses observed following FMT. While small sample sizes preclude attribution or causality, this report offers a rare window into the host T cell response following FMT, particularly of T regulatory and MAIT cells which have been previously shown to be altered in UC patients [34–36]. Our data from peripheral blood samples support the notion that MAIT cell populations traffic to the mucosal surface of UC patients where they may modulate microbial stimuli [37–41]. The observed decrease in IL-17A and IFNγ MAIT cell positivity observed in subjects with improved clinical scores further supports a potential role for MAIT cells in the response of UC patients to FMT and warrants further study. Ultimately, an enhanced understanding of the defining clinical and immunological characteristics of UC patients poised to respond favorably to FMT may aid in moving FMT and other microbially-based therapies forward.

FMT can significantly alter the gut microbiota of recipients [9, 18]. We show that, by 2 weeks after FMT induction, the gut microbiota of UC patients is highly correlated with that of donors, and that these changes persist up to 20 weeks. cFMT appears to reinforce the donor convergence period, extending the currently reported interval by 4 weeks [11–14]. It does not, however, appear that the degree of donor correlation is an indicator of positive FMT clinical effects as no difference in donor convergence was observed between potential FMT “responders” and “non-responders” in our small sample.

A disconnect between donor convergence and clinical response has also been reported in individuals treated for recurrent *C. difficile*. These patients demonstrate variable patterns of donor microbial divergence following FMT with little correlation to clinical outcome. This observation suggests that a more granular and complex understanding of FMT-induced microbial changes is desirable [17, 42]. While the ultimate durability of FMT-induced changes in the microbiota is unknown, studies in recurrent *C. difficile* patient populations have shown high donor correlations to persist for greater than 1 year [9, 17]. The durability of FMT-induced microbial changes in IBD populations is less well established. In prior studies using serial endoscopic or enema-based regimens, persistent microbiota changes have been detected for up to 16 weeks in UC patients [11–14]. The only report to our knowledge of microbial changes following oral FMT monotherapy in UC patients showed no differences in alpha diversity nor evidence of donor convergence [27], raising the possibility that large volume FMT induction may be necessary to achieve initial community-level donor convergence.

As a small pilot trial, this study was not powered to statistically demonstrate a difference in clinical outcome and thus preliminary observations must be replicated in larger trials. Despite its small size however, the observed changes were consistent with previously published clinical studies and immunologic observations recapitulated those of others [29, 36]. Future studies should evaluate the different FMT dosing ranges and regimens in the treatment of UC in both induction and maintenance phase. Further inquiry should also strive to include immune profiling data from mucosal tissues at the site of disease activity to better understand FMT-induced immunologic responses to alterations in the colonic microbiome.

## Conclusions

Oral cFMT formulations are likely to gain acceptance by some individuals as a therapeutic alternative in UC, and may enhance the potential for longterm microbially-based treatment strategies. To date, scientific data regarding the feasibility, safety and efficacy of these regimens are lacking. This study provides initial evidence that cFMT is a safe, and well-tolerated adjunctive mechanism by which to support and extend FMT-induced shifts in the gut microbiota of UC patients. Future studies should address optimal dose and dosing strategies of cFMT as well as temperature-stable formulations.

## Supplementary Information

**Additional file 1**. Clinical study flow diagram showing the overall study design.

**Additional file 2**. Supplemental materials and methods section.

**Additional file 3**. CONSORT reporting checklist for randomized controlled clinical trials.

**Additional file 4**. Clinical study protocol.

## Data Availability

The 16S datasets generated and analysed during the current study are available in the NCBI’s SRA database repository, (BioProject PRJNA475599). The clinical datasets generated and analysed during the current study are not publicly available due privacy issues, but are available from the corresponding author on reasonable request.

## Abbreviations

AE: Adverse event
BMI: Body Mass Index
CD: Crohn’s disease
cFMT: Capsule FMT
CI: Confidence interval
CONSORT: Consolidated Standards of Reporting Trials
CRP: C-reactive protein
FMT: Fecal microbiota transplantation
IBD: Inflammatory Bowel Disease
IBDQ: Inflammatory Bowel Disease Questionnaire
IFNγ: Interferon gamma
IL-10: Interleukin-10
IL-17A: Interleukin-17A
IND: Investigational New Drug
MAIT: Mucosal associated invariant T cells
PBMC: Peripheral blood mononuclear cells
RB: Rectal bleeding
RR: Relative risk
SF: Stool frequency
Treg: T regulatory cells
UC: Ulcerative colitis
UCEIS: Ulcerative Colitis Endoscopic Index of Severity
UVMMC: University of Vermont Medical Center
