# Supplementary figures and images for: Daily, oral FMT for long-term maintenance therapy in ulcerative colitis: results of a single-center, prospective, randomized pilot study
Source: BMC Gastroenterol. 2021 Jul 8;21:281. doi: 10.1186/s12876-021-01856-9 (PMC8268596; doi:10.1186/s12876-021-01856-9)

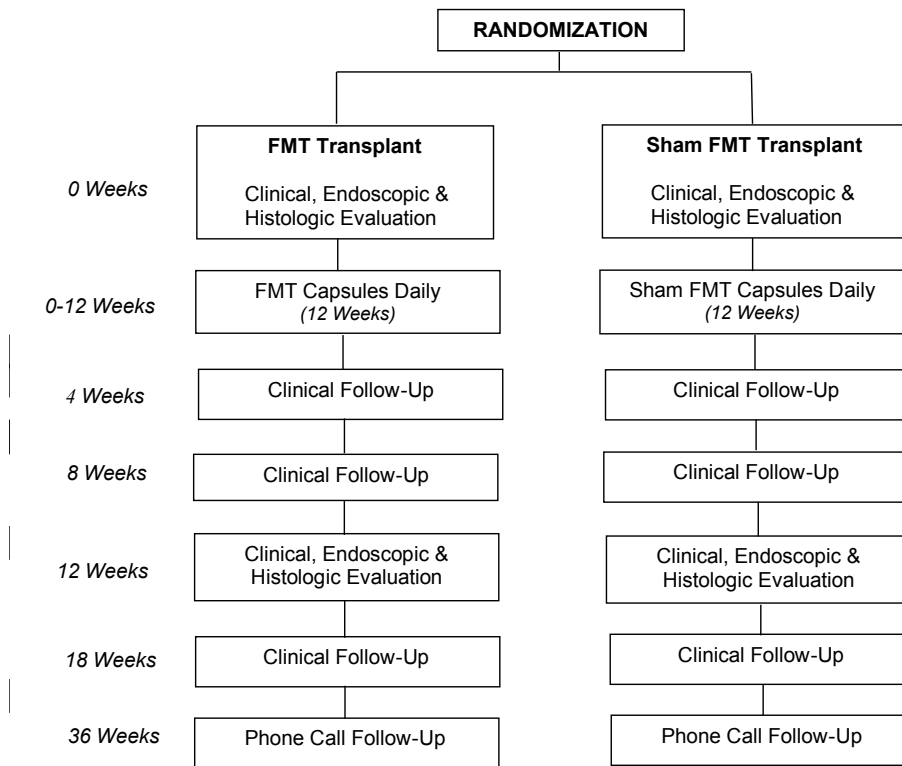

Supplement: Supplementary file 1 — Additional file 1. Clinical study flow diagram showing the overall study design. [file 12876_2021_1856_MOESM1_ESM.pdf]
