# Supplementary material for: Daily, oral FMT for long-term maintenance therapy in ulcerative colitis: results of a single-center, prospective, randomized pilot study
Source: BMC Gastroenterol. 2021 Jul 8;21:281. doi: 10.1186/s12876-021-01856-9 (PMC8268596; doi:10.1186/s12876-021-01856-9)
Supplement: Supplementary file 2 — Additional file 2. Supplemental materials and methods section. [file 12876_2021_1856_MOESM2_ESM.docx]

**SUPPLEMENTAL PROCEDURES (MATERIALS & METHODS)**

*Human Samples and Cell isolation*

Peripheral heparinized blood samples were obtained from study subjects at weeks 0, 4, 8 and 12. Blood samples were maintained at room temperature and processed within 2 hours of collection.  PBMCs were isolated by Ficoll (Histopaque, Sigma Life Science, United Kingdom) gradient centrifugation, resuspended in phosphate-buffered saline with 2 mM EDTA and 0.02% bovine serum albumin. Following multiple rinses, PBMCs were stored in 10% DMSO with 90% fetal calf serum at minus 80°C until analysis.

*Flow Cytometry and Intracellular cytokine staining*

Upon thawing, PBMCs and intestinal biopsy mononuclear cells were resuspended in prewarmed complete medium (RPMI 1640 (Mediatech, Inc., Herndon, VA, USA), 2.5 mg/ml glucose (Sigma, St. Louis, MO), 10 mg/ml folate (Invitrogen, Carlsbad, CA), 110 μg/ml pyruvate (Invitrogen), 5 × 10^–5^M 2-mercaptoethanol (Sigma), 292.3 μg/ml glutamine (Invitrogen), 100 units/ml penicillin-streptomycin (Invitrogen), and 5% bovine calf serum) and allowed to rest overnight. The following morning, cells were washed in medium (RPMI 1640 (Mediatech, Inc., Herndon, VA, USA) plus 100 units/ml penicillin-streptomycin (Invitrogen), and 5% bovine calf serum) with the addition of Benzonase Nuclease (339U/mL, Novagen Millipore, Billerica, MA). Cells were then stimulated with phorbol myristate acetate (50 ng/ml, Sigma, St. Louis, MO) and ionomycin (500 ng/ml, Sigma, St. Louis, MO) in the presence of brefeldin A (GolgiPlug, used per manufacture recommendations, BD Biosciences, San Jose, CA), and monensin (GolgiStop, used per manufacture recommendations, BD Biosciences, San Jose, CA) for 5 hours at 37**°**C with 7% CO_2_.  A negative control for intracellular cytokine staining ommitting brefeldin and monenson was included. Unstimulated samples were resuspended for the same amount of time in the same conditions in 100 μL of the same media with the omission of Phorbol myristate acetate and Ionomycin. After 5 hours, 1.5 mL of complete media was added to samples and they were allowed to rest overnight at 4**°**C. The following day, samples were washed, stained with a near-IR fluorescence reactive dye (Life technologies, Eugene, OR), followed by an Fc blocking step (Fc Block, BioLegend, Human TruStain FcX, San Diego, CA), followed by a surface antibody mix which included: TCR αβ (BioLegend, Alexa Fluor 488, clone IP26), CD45 (BioLegend, Alexa Fluor 700, clone 2D1), CD8 (BD Horizon, BUV395, clone RPA-T8), CD4 (BioLegend, BV510, clone RPA-T4), CD13 (BioLegend, PE/Dazzle, clone WM15), TCR γδ (BioLegend, BV421, clone B1), CD25 (BioLegend BV650, clone BC96), hMR1 (NIH tetramer facility APC, 5-OP-RU 2017-04-07, Atlanta, GA). Antibodies were mixed using a brillant stain buffer (BD Horizon,San Jose, CA). After incubation and washings, cells were fixed and permeabilized (BD Bioscience Fixation/Permeabilization Solution Kit, San Jose, CA). Following an additional Fc Blocking step (Fc Block (BioLegend, Human TruStain FcX, San Diego, CA), intracellular cytokine staining was performed using the following antibodies: IL-17 (BioLegend PerCP/Cy5.5, clone BL168), IL-10 (BioLegendPE/Cy7, clone JES3-9D7), IFN-γ BioLegend PE, clone B27).

 After washing, cells were fixed with freshly made 1% (v/v) methanol-free formaldehyde (Ted Pella Inc., Redding, CA) in PBS/1% BSA. Samples were run the following day within the UVM-LCOM Flow Cytometry and Cell Sorting facility on an LSRII (BD Biosciences, San Jose, CA). Two hundred thousand events were collected from all samples unless limited by sample concentration, in which case as many events as possible were collected.

Analysis was performed using FlowJo software (v 10.4.1, Tree Star, Inc., Ashland, OR). Cells were first gated for their uptake of near-IR fluorescence reactive dye (vs. FSC-A) to determine live versus dead cells.  Only the live population was then used to gate on lymphocytes (FSC-A vs SSC-A). Non-lymphoid cells (monocytes, macrophages, granulocytes) were then excluded (CD13 vs. CD14) and gating for singlets performed (FSC-A vs FSA-H). The resultant population was further clarified by an additional gating step again using uptake of near-IR fluorescence reactive dye (vs. CD45) to confirm hematopoietic origin. This live lymphoid population (Live, singlet, CD13-, CD14-, CD45+) was taken for all downstream analysis.  TCR-alpha beta and TCR-γδ expression was next determined from this population.  Within the TCR- αβ^+^ gate, MR1 expression was determined. CD4 or CD8 surface expression was then determined within the MR1^+^and MR1^-^gates. TCR αβ^+^CD4^+^MR1^+^cells were defined as Mucosal Associated invariant T cells (MAIT) in the context of this paper. TCR- αβ^+^CD4^+^MR1^-^cells were further characterized by their expression of CD25, with CD25^hi^ cells defined as T-regulatory cells in the context of this paper. The percentage of cells positive for all combinations of intracellular markers were then determined.

The percentage of TCR- γδ^+^ and TCRαβ^+^ were then calculated out of the total live lymphoid population (Live, singlet, CD13-, CD14-, CD45+) and the following cell populations out of  the total TCRαβ^+^population: TCRαβ^+^CD8^+^, TCRαβ^+^CD4^+^CD25^-^, TCRαβ^+^CD4^+^CD25^+ (^T regulatory cells), TCR αβ^+^MR1^+^(MAIT cells). The percentage of cells positive for any combination of intracellular marker expression was then determined within each of these terminal populations. IFNγ expression was first assessed (IFNγ vs. CD45).  IFNγ^+^and IFNγ^–^ populations were each then assessed for IL-10 and IL-17A expression.

Prior to analysis, a flow cytometry protocol was optimized by first using peripheral blood samples and then mucosal samples from surgically obtained specimens. Fluorescence Minus One (FMO) controls were performed prior to running patient samples. The following filter configuration was used on an LSRII (BD Biosciences, San Jose, CA): Violet Laser with PMT A (BP emission filter 660/20), PMT B (BP emission filter 525/50, PMT C (BP emission filter 450/50), UV laser with PMT A (BP 740/35), and PMY B (BP 379/28).

*Fecal Microbiota Profiling of Stool Samples*

Stool samples were obtained weekly throughout the study period, beginning prior to antibiotic pretreatment and ending at 18-weeks follow-up. Patients were provided with home stool collection kits and instructed to collect samples weekly at roughly the same time each day. Approximately 1g of stool was dissolved in 5ml RNALater (Ambion, Inc.) pre-aliquoted into Para-Pak vials (Meridian Biosciences, Inc.) and transported at room temperature to the study team by US mail. Upon receipt, samples were transferred to -80°C until analysis.

Prior to DNA extraction, samples were thoroughly washed with PBS buffer.  For DNA extraction, the MoBio Powersoil 96 kit (now Qiagen Cat No./ID: 12955-4) was used with minor modifications. All samples were thawed on ice and between 50 ug – 150ug of homogenized stool was transferred to the MoBio High Throughput PowerSoil bead plate (12955-4-BP), covered with the Square Well Mat and stored at -20°C overnight prior to being processed according to the manufacturer’s protocol.  16S rRNA gene libraries targeting the V4 region of the 16S rRNA gene were prepared by first normalizing template concentrations and determining optimal cycle number by way of qPCR.  Two 25 uL reactions for each sample were amplified with 0.5 units of Phusion with 1X High Fidelity buffer, 200 μM of each dNTP, 0.3 μM of 515F (5’AATGATACGGCGACCACCGAGATCTACACTATGGTAATTGTGTGCCAGCMGCCGCGGTAA-3’) and 806rcbc0 (5’CAAGCAGAAGACGGCATACGAGATTCCCTTGTCTCCAGTCAGTCAGCCGGACTACHVGGGTWTCTAAT-3’).  0.25 µL 100x SYBR were added to each reaction and samples quantified using the formula 1.75^(deltaCt).  To ensure minimal over-amplification, each sample was diluted to the lowest concentration sample, amplifying with this sample optimal cycle number for the library construction PCR.  Four 25µL reactions were prepared per sample with master mix conditions listed above, without SYBR.  Each sample was given a unique reverse barcode primer from the Golay primer set^67,68^. Replicates were then pooled and cleaned via Agencourt AMPure XP-PCR purification system.  Purified libraries were diluted 1:100 and quantified again via qPCR (Two 25µL reactions, 2x iQ SYBR SUPERMix (Bio-Rad, REF: 1708880 with Read 1 (5’-TATGGTAATT GT GTGYCAGCMGCCGCGGTAA-3’), Read 2 (5’-AGTCAGTCAG CC GGACTACNVGGGTWTCTAAT-3’).  Undiluted samples were normalized by way of pooling using the formula mentioned above.  Pools were quantified by Qubit (Life Technologies, Inc.) and normalized into a final pool by Qubit concentration and number of samples.  Final pools were sequenced on an Illumina MiSeq 300 using custom index 5’-ATTAGAWACCCBDGTAGTCC GG CTGACTGACT-3’ and custom Read 1 and Read 2 mentioned above.  Raw sequence reads were then processed and OTU calling performed. First, adapter sequences from raw paired-end 16S rRNA Illumina sequencing reads were removed using Cutadapt. The paired-end reads were then trimmed, denoised, and merged to a common length of 233 bases.  Dereplicated reads were clustered into OTUs using the Qiime2 – dada2 pipeline. Samples with fewer than 4000 reads (3 out of 222) were discarded from the analysis.  Statistical tests of differential abundance, microbial alpha diversity (Shannon Index) and Jennsen-Shannon divergence indices from both donor samples and baseline samples for each individual were calculated. Correlations with other investigational measures, notably immunologic profiles, were also performed.
