# Supplementary material for: Daily, oral FMT for long-term maintenance therapy in ulcerative colitis: results of a single-center, prospective, randomized pilot study
Source: BMC Gastroenterol. 2021 Jul 8;21:281. doi: 10.1186/s12876-021-01856-9 (PMC8268596; doi:10.1186/s12876-021-01856-9)
Supplement: Supplementary file 4 — Additional file 4. Clinical study protocol. [file 12876_2021_1856_MOESM4_ESM.pdf]

Fecal Microbiota Transplantation and Supplementation in Refractory Ulcerative Colitis

Serial 00009

Peter L. Moses, MD
Professor, Department of Gastroenterology
University of Vermont Medical Center

FMT Protocol Version and Consent Tracker			
Item	***Submission Date to IRB***	***Protocol Version and Date***	***ICF Version and Date***
Initial Submission to IRB	02Feb2015	(V1) 21Jan2015	(V1) 17Jan2015
Modifications to Secure Approval	31March2015	(V1) 21Jan2015	(V1) 17Jan2015
FDA Revisions for Clinical/Non-Clinical Hold	02June2015	Serial 0001 (INDA)	(V2)30May2015
IRB Revisions from FDA Modifications (1)	16July2015	(V2) 30May2015	(V2)30May2015
IRB Revisions from FDA Modifications (2)	07August2015	(V3) 07August2015	(V2) 07August2015
Amendment 1	28August2015	(V4) 26August2015	(V3) 26Augsut2015
Amendment 1 (IRB Modifications)	07October2015	(V4) 26August 2015	(V3) 26Augsut2015
FINAL IRB APPROVAL		(V4) 26August 2015	(V3) 26Augsut2015
Amendment 2 (FDA Serial 00003)	12December2015	(V5) 27November2015	(V4) 27November2015
Amendment 3 (FDA Serial 00004)	09October2016	(V6) 07October2016	(V5) 07October2016
Amendment 3.1 (FDA Serial 00004)	02November2016	(V7) 01November2016	(V5) 07October2016
Amendment 3.2 (FDA Serial 00005)	24January2017	(V8) 24January2017	Pilot/Controls ICF Only: (V4) 24January2017
Amendment 4 (FDA Serial 00006)	16MAY2017	(V9) 15MAY2017	(V6) 15MAY2017
Amendment 5 (FDA Serial 00007)	28AUG2017	(V10) 28AUG2017	ICF: (V6)15MAY2017 Pil/Con: (V4) 24JAN2017
Amendment 6 (FDA Serial 00008)	06OCT2017	(V11 03OCT2017)	ICF: (V6)15MAY2017 Pil/Con: (V4) 24JAN2017
**Amendment 7 (FDA Serial 00009)**	**18MAY2018**	**(V12 18MAY2018)**	ICF: (V6)15MAY2017 Pil/Con: (V4) 24JAN2017

3.1. Introductory Statement 4
3.1.1. Name of the Drug and All Active Ingredients 5
3.1.2. Pharmacological Class of the Drug 5
3.1.3. Structural Formula of the Drug 5
3.1.4. Formulation of the Dosage Forms to be Used 5
3.1.5. Route of Administration 6
3.1.6. Objectives and Duration of the Proposed Clinical Investigations.... 6
3.2. Summary of Previous Human Experience 6
3.3. Status of Drug in Other Countries 7
3.4. References....7
4. General Investigational Plan 8
4.1. Rationale 8
4.2. Indication to be Studied 9
4.3. General Approach for Evaluation of Treatment 9
4.4. Collection of Pliot Tissue and Control Tissue....9
4.5. Description of First Year Trial(s)....10
4.6. Number of Subjects to be Evaluated 11
4.7. Drug Related Risks 11
5. Investigator Brochure 12
5.1. Drug Substance and Formulation 12
5.2. Pharmacological and Toxicological Effects of the Drug 12
5.3. Safety and Effectiveness of the Drug....12
5.4. Risks and Side Effects 12
5.4.1. Potential risks to subjects....12
5.4.2. Potential risks to donors....13
5.4.3. Protection agaist risks to subjects and donors....13
5.4.4. Saftey moniotoring protocol....15
5.4.5. Data moniotring plan....16
5.4.6. Education training....16
6. Protocol 17
6.1. Study Protocol....17
6.2. Informed Consent 26
6.3. Investigator and Facilities Data 26
7. Chemistry, manufacturing and control information....26
7.1. drug substance 26
7.1.1. The name and address of the drug manufacturer 26
7.2. Drug product 27
7.2.1. The name and address of the drug product manufacturer 27
7.3. Placebo 27
7.4. Labeling 27
7.5. Environmental assessment 28
8. Pharmacology and toxicology information....28
8.2. Toxicology 28
8.3. Statement of compliance with glp 28
9. Previous human experience 28
9.1 previous experience with treatement of clostridum difficile infection 28
9.2 previous experience with treatement of inflammatory bowel disease....29
10. Aditional information 30
10.1. Drug dependence and abuse potential 30
10.2. Radioactive drugs 30
10.3. Pediatric studies 30
11. APPENDICES 31

3. Introduction

3.1 Introductory Statement

Inflammatory Bowel Disease (IBD) is a chronically relapsing, complex disease likely related to both environmental and genetic factors. The interaction between host genetic factors, combined with a dysregulated immune response to bacterial antigens has been hypothesized in the pathogenesis of IBD. Already, several lines of evidence strongly support the link between the immune response generated by the intestinal microbiome and the development of IBD. Animal studies have already demonstrated the pivotal role of enteric bacteria in the development of colitis.[(1)] Studies have also documented decreased biodiversity and an imbalance between protective and pathogenic microbes in the development of IBD. [(2,3)] Even in clinical practice, reinfusion of luminal contents into previously bypassed colonic segments have been shown to induce colitis- thus suggesting that the direct interaction of the gut microbiota with colonic mucosa is, at least, partly responsible for relapsing inflammatory bowel disease. [(4,5)]

Our understanding of the specific microbes involved in the pathogenesis of IBD is limited, partly due to our inability to completely characterize the human microbiome. It is estimated that only 5-30% of the human microbiome is able to be cultured. [(6)] Newer technologies in gene sequencing allow for improved characterization of the microbial ecosystem present within the human gastrointestinal tract. With new developments in genomic sequencing, more precise comparisons between the gut microbiome of healthy and diseased individuals is now possible.

The microbiome in patients with IBD has consistently been shown to be less diverse with decreased populations of Bacteroidetes and Firmicutes, two important bacterial phyla associated with normal gut function. [(7,8)] While it is not known if these differences are related to the underlying cause of IBD or simply a result of mucosal inflammation, mouse models have shown that bacteria within both Bacteroidetes and Fimicutes phyla are capable of activating regulatory T cells. These findings suggest that manipulation of the microbiota may be effective in treating IBD.

Fecal Microbiota Transplant (FMT) or fecal bacteriotherapy refers to the infusion of homogenized fecal suspension from a healthy individual into the GI tract of another individual. Metagenomic sequencing has demonstrated that FMT can alter the bacterial composition of the human gut, leading to a durable microbiome in the recipient that reflects the composition of the donor. [(9)] By taking advantage of this microbial shift, FMT is now a recognized treatment for recurrent *Clostridium difficile* colitis when standard antibiotic therapy fails. A prospective randomized trial showed FMT to have a success rate of 94% in treating recurrent *C. difficile*.[(10)] Another multi-center trial of FMT delivered by colonoscopy for recurrent *C. difficile* infection reported a 91% primary cure rate and a 98% secondary cure rate. [(11)]

Standard treatment for IBD using oral 5-ASA, immunomodulators and biologic agents often results in incomplete remissions and ongoing symptoms. In patients who continue to be symptomatic despite aggressive standard medical treatment for colitis, altering the bacterial antigens in the gut which bear on immune regulation is an intriguing option for additive treatment.

There have been several case series and cohort studies of IBD patients achieving clinical remission following FMT. [(12,13,14)] A systematic review conducted by Anderson et al[(15)] reported that FMT could achieve clinical remission in 63% of IBD patients. More recently, in the largest systematic review and meta-analysis of FMT in IBD, roughly 45% of IBD patients achieved clinical remission during follow-up. [(16)]

While these results have been praised by many in the medical community, the known efficacy of FMT in IBD is limited by the lack of prospective, randomized controlled trials with strict protocols. Our study

will investigate the utility of a novel FMT protocol with administration of fecal microbiota by colonoscopy as induction treatment in conjunction with prolonged oral supplementation of encapsulated bacteriotherapy as maintenance treatment. The recording of symptoms, endoscopic findings, histologic activity and metagenomic data will objectively elucidate the effect of FMT in the ulcerative colitis population.

3.1.1 Name of the Drug and All Active Ingredients

Fecal Microbiota Preparation

Frozen human fecal microbiota (active ingredient); USP Glycerol (.99% glycerol in 1% water, excipient); Cocoa Butter (excipient); acid-resistant HPMC Capsules (excipient)

3.1.2 Pharmacological Class of the Drug

Experimental - Biological Product

3.1.3 Structural Formula of the Drug

Human Stool

3.1.4 Formulation of the Dosage Forms to be Used

The initial fecal microbiota transplantation delivered by colonoscopy will contain 120 mL of liquid fecal microbiota preparation from four 30 mL bottles. The fecal microbiota preparation is synthesized from human feces collected from healthy individuals. The sample is deposited into a specimen collection container and then properly sealed into one resealable LDPE plastic bag (Ri-Pac 2GN or similar) as secondary containment. Collected samples are transferred to a UV-sanitized biosafety cabinet that is exclusively dedicated to sample processing and that is cleaned with a sporicidal disinfectant. Within the biosafety cabinet, the stool is transferred to a sterile filter bag. An autoclaved or sterile-filtered diluent consisting of 12.5% glycerol and a normal saline buffer is added to the filter bag at a ratio of 2.5 milliliters of buffer per gram of stool. The filter bag is then sealed and introduced to secondary containment within a homogenizer blender for 120 seconds to homogenize the stool and buffer (or longer if needed to obtain full homogenization). The sample is then aliquoted into sterile 30 milliliter bottles using sterile, disposable serological pipettes. The bottles are then capped and frozen immediately at -20°C. Bottles are transferred from -20°C to -80°C at the end of each production day. Caps are sealed with tamper-evident, perforated PVC shrink bands to provide an additional level of containment and ensure samples are not altered during storage and distribution.

The oral preparation will consist of an autoclaved diluent consisting of 80% cocoa butter and 20% glycerol that is added to the filter bag of stool at a ratio of 1g stool:1mL diluent. Samples will be homogenized to form a microbial emulsion matrix (MEM) and aliquoted into a sterile reservoir using sterile, disposable serological pipettes. During encapsulation, 750 µL of the MEM material (kept at 37ºC) will be micropipetted into a Size 00 acid-resistant, HPMC colored capsule (Capsugel, White #G60CS000753). The capsules will be closed and stored in sterile bottles at -80ºC. At time of dosing, capsules will be transferred to blister packaging. Small plastic bags will contain 7 individually-wrapped blister packaged capsules for each week of treatment.

Preparations will be stored at - 80°C and shipped on dry ice with an enclosed temperature monitor to ensure that the product has remained frozen during transit. Upon arrival, the product will be immediately transferred to a standard -20°C (-4°F) freezer. The liquid preparation will be thawed immediately prior to colonoscopy, which will be performed within 4 hours of thawing. The capsule preparations will be given to subjects in 6-week aliquots (42 capsules) inside of a Styrofoam cooler with an icepack and dry ice. Subjects will be instructed to keep the coolers inside their home freezers with the ice pack. Maintenance of the capsules inside coolers with icepacks will mitigate the effect of temperature fluctuations within home freezers on the capsules.

3.1.5. Route of Administration

Initial fecal microbiota transplantation in the "induction" cohort will be delivered in liquid form to the terminal ileum and colon via colonoscopy. Any supplemental administration of fecal microbiota during the "maintenance" window will be delivered orally in gelatin capsules.

3.1.6. Objectives and Duration of the Proposed Clinical Investigations

Primary objective: To assess the safety and efficacy of FMT therapy in patients with mild-moderate ulcerative colitis despite standard medical treatment measured in terms of patient symptoms, endoscopic appearance of colon, histologic grade from colonic biopsies, and biologic markers of inflammation (CRP fecal calprotectin and fecal lactoferrin).

Secondary objective: To assess changes in microbial and metagenomic profiles pre and post- FMT therapy.

Tertiary objective: To evaluate possible mechanisms responsible for FMT's effect. These include immunophenotypic profiling of the inflammatory response, serotonin signaling markers, and serum metabolites pre and post- FMT therapy.

Total duration of FMT therapy will be 12 weeks. Subjects will be randomized to either a real or sham FMT arm. Subjects assigned to the control arm will receive sham FMT by colonoscopy (brown-colored placebo saline followed by 12 weeks of 1 capsule per day PO intake of sham FMT capsules (brown-colored placebo saline within gelatin capsules). Subjects assigned to the experimental arm will receive real FMT by colonoscopy followed by 12 weeks of 1 capsule per day PO intake of real FMT capsules.

3.2 Summary of Previous Human Experience

Fecal microbiota transplantation has already been used with highly successful cure rates and low complication rates in treating recurrent infection related to *Clostridium difficile*. A robust systematic review and meta-analysis of 11 observational studies with a total of 273 CDI patients did not report any adverse events attributable to the FMT material in variable follow-up from 3 weeks to 8 years.[17] However, 3 studies that used upper gastrointestinal FMT indicated that the nasogastric/nasojejunal tube itself could not be ruled out in contributing to a suspected adverse event: upper gastrointestinal bleed, peritonitis, and possibly enteritis.[17] A multi-center retrospective review (n=77) reported no definite FMT adverse events in follow-up between 3-68 months (mean 17 months). The authors did report 4 patients developing diseases of potential interest after FMT (peripheral neuropathy, Sjogren's disease, idiopathic thromobocytopenic purpura, rheumatoid arthritis) and 7 non-FMT related deaths. Interestingly, the study noted post-FMT improvement in a patient with pre-existing allergic sinusitis and one with "arthritis", with the type unreported.[11]

Safety outcomes for FMT treatment of CDI have also been reported in immunocompromised patients in a multi-center retrospective review (n=80).[18] In this special group, 12 patients (15%) had a serious adverse event within 12 weeks of FMT, of which 10 were hospitalized. Specifically, 2 deaths occurred within 12 weeks, one a result of aspiration at the time of colonoscopy for FMT, and the other unrelated to FMT (progressive pneumonia). There were 3 deaths reported 6 months after FMT due to chronic, progressive medical conditions unrelated to FMT. There was another procedure-related complication as a subject sustained a superficial mucosal tear during colonoscopy. Importantly, no patients experienced an infection definitively ascribed to FMT, although 2 subjects sustained unrelated infections (influenza, catheter line infection). Self-limited diarrheal illness occurred in 5 patients but no causal organism was identified.[18]

The use of fecal microbiota transplantation in managing inflammatory bowel disease is still poorly understood. In the largest review and meta-analysis of fecal microbiota transplantation in patients with inflammatory bowel disease, 45% (54/119) patients achieved clinical remission during follow-up, with mucosal healing demonstrated in 12/16 (75%) case study patients.[(15)] Separately, the largest randomized controlled trial to date demonstrated endoscopic remission in 23% of patients with active ulcerative colitis who were given fecal microbiota therapy via enema, compared to only 7% who were given placebo. [(19)]

The safety of fecal microbiota transplantation in inflammatory bowel disease has been extrapolated from several cohort studies. Overall, fecal microbiota transplantation is very well tolerated and has proven to be safe in patients with *Clostridium difficile*. In the largest review and meta-analysis of fecal microbiota transplantation in patients with inflammatory bowel disease, 11/79 patients (15%) experienced fever, abdominal tenderness, rigors, CRP elevation or the combination of variables.[15] Fever severity ranged from self-limiting to requiring acetaminophen therapy with resolution and ranged from hours to days after the procedure. No major adverse events have been reported in the largest randomized controlled trial involving nearly 30 patients with ulcerative colitis receiving fecal microbiota transplantation. [19]

3.3 Status of Drug in Other Countries

The overall safety and efficacy of fecal microbiota transplantation is currently under investigation in other countries.

3.4 References

1. Saleh M, Elson CO. Experimental inflammatory bowel disease: insights into the host-microbiota dialog. Immunity 2011; 34: 293-302
2. Qin J, et al. A human gut microbial gene catalogue established by metagenomic sequencing. Nature 2010; 464:59-65
3. Manichanh C, Borruel N, Casellas F, Guarner F. The gut microbiota in IBD. Nat Rev Gastroenterol Hepatol 2012; 9: 599-608
4. Fichera A, McCormack R, Rubin MA, Hurst RD, Michelassi F. Long-term outcome of surgically treated Crohn's colitis: prospective study. Dis Colon Rectum 2005; 48: 963-969
5. Rutgeerts P, Goboes K, Peeters M, Hiele M, Penninckx F, Aerts R, Kerremans R, Vantrappen G. Effect of faecal stream diversion on recurrence of Crohn's disease in the neoterminal ileum. Lancet 1991; 338: 771-774
6. Eckburg PB, Bik EM, Bernstein CN, Purdom E, Dethlefsen L, Sargent M, Gill SR, Nelson KE, Relman DA. Diversity of thehuman intestinal microbial flora. Science 2005; 308: 1635-1638
7. Morgan XC, Tickle TL, Sokol H, Gevers D, Devaney KL, Ward DV, Reyes JA, Shah SA, LeLeiko N, Snapper SB, Bousvaros A, Korzenik J, Sands BE, Xavier RJ, Huttenhower C. Dysfunction of the intestinal microbiome in inflammatory bowel disease and treatment. Genome Biol 2012; 13: R79
8. Frank DN, St Amand AL, Feldman RA, Boedeker EC, Harpaz N, Pace NR. Molecular-phylogenetic characterization of microbial community imbalances in human inflammatory bowel diseases. Proc Natl Acad Sci USA 2007; 104: 13780-13785
9. Grehan MJ, Borody TJ, Leis SM, Campbell J, Mitchell H, Wettstein A. Durable alteration of the colonic microbiota by the administration of donor fecal flora. J Clin Gastroenterol 2010; 44: 551-561
10. van Nood E, Vrieze A, Nieuwdorp M, Fuentes S, Zoetendal EG, de Vos WM, Visser CE, Kuijper EJ, Bartelsman JF, Tijssen JG, Speelman P, Dijkgraaf MG, Keller JJ. Duodenal infusion of donor feces for recurrent Clostridium difficile. N Engl J Med 2013; 368: 407-415 [PMID: 23323867 DOI: 10.1056/NEJMoa1205037]
11. Brandt LJ, Aroniadis OC, Mellow M, Kanatzar A, Kelly C, Park T, Stollman N, Rohlke F, Surawicz C. Long-term followup of colonoscopic fecal microbiota transplant for recurrent Clostridium difficile infection. Am J Gastroenterol 2012; 107: 1079-1087 [PMID: 22450732 DOI: 10.1038/ajg.2012.60]
12. Bennet JD, Brinkman M. Treatment of ulcerative colitis by implantation of normal colonic flora. Lancet 1989; 1: 164
13. Borody TJ, Warren EF, Leis S, Surace R, Ashman O. Treatment of ulcerative colitis using fecal bacteriotherapy. J Clin Gastroenterol 2003; 37: 42-47
14. Kunde S, Pham A, Bonczyk S, Crumb T, Duba M, Conrad H, Cloney D, Kugathasan S. Safety, tolerability, and clinical response after fecal transplantation in children and young adults with ulcerative colitis. J Pediatr Gastroenterol Nutr 2013; 56: 597-601
15. Anderson JL, Edney RJ, Whelan K. Systematic review: faecal microbiota transplantation in the management of inflammatory bowel disease. Aliment Pharmacol Ther 2012; 36: 503-516
16. Colman RJ, Rubin D. Fecal microbiota transplantation as therapy for inflammatory bowel disease: A systematic review and meta-analysis. Journal of Crohn's and Colitis 2014; 8: 1569-1581

17. Kassam, Z., Lee, C. H., Yuan, Y. & Hunt, R. H. Fecal microbiota transplantation for Clostridium difficile infection: systematic review and meta-analysis. Am. J. Gastroenterol. 108, 500–508 (2013).
18. Kelly, C. R. et al. Fecal Microbiota Transplant for Treatment of Clostridium difficile Infection in Immunocompromised Patients. Am. J. Gastroenterol.109, 1065–71 (2014).
19. Moayyedi P, Surette M, Wolfe M, Taraschi R, Kim P, Libertucci J, et al. A randomized, placebo controlled trial of fecal microbiota therapy in active ulcerative colitis. Gastroenterology 2014; 146: S-159

4.GENERAL INVESTIGATIONAL PLAN

4.1 Rationale

The interaction of microbes with mucosal immune cells in the gastrointestinal tract is thought to have a major role in priming and regulating global host immunity. Recently it has been appreciated that aspects of health depend on a beneficial host–microbe interaction. The enteric environment is known as the microbiome (MB) and alteration of the MB is now thought to play a role in the development of UC [20]. It has been proposed that changes in the microbiota composition are responsible for activating a pathogenic immune response in IBD patients.

We hypothesize that disruption of the host microbiome (antibiotic pretreatment) followed by prolonged exposure to a diverse microbiota is needed for a durable "probiotic" effect, eventually restoring a healthy host-microbe interaction in UC patients. In restoring this flora, the concept of combining administration of fecal microbiota via colonoscopy and via prolonged oral intake is novel.

The decision to pretreat patients with antibiotics reflects the opinion of experts in the field of the microbiome. Different case reports and cohort studies have used various FMT protocols, but the importance of removing resident bowel flora prior to fecal transplant has been emphasized by many authors. Thomas Borody, a pioneer in the field of FMT, originally observed that patient symptoms transiently diminished after gastrointestinal lavage prior to colonoscopy and following the use of antibiotics (1). In support of this, Borody published a case series of patients with UC in 2003, all of whom responded to FMT following pre-treatment antibiotics and polyethylene gycol lavage (GoLYTELY®)(2). "Resetting" the host's dysbiotic MB with antibiotics for one week followed by polyethyelene glycol x1 may potentiate the therapeutic effects of FMT therapy and explain why other studies forgoing pretreatment have not been as effective.

Importantly, antibiotics have been shown to be efficacious in the general treatment of UC. Even outside of a clinical trial, administration of ciprofloxacin and metronidazole can be prescribed for patients with IBD. A meta-analysis published in 2011 with 9 randomized control trials showed a statistically significant benefit for antibiotics inducing remission in UC (3) . This makes teleological sense given the belief that IBD results, in part, from an aberrant immunological reaction to gut microbiota in susceptible hosts. Therefore, in order for FMT to be the only treatment variable, subjects in both the control and the experimental arm will undergo identical pretreatment antibiotic regimens

It is important to note that in order to demonstrate effectiveness of FMT against UC beyond this Phase 1 study will require evaluation of added benefit, if any, of pretreatment antibiotics.

References

1.Borody TJ, George L, Andrews P, et al. Bowel-flora alteration: a potential cure for inflammatory bowel disease and irritable bowel syndrome? Med J Aust. 1989;150(10):604
2.Borody TJ, Warren EF, Leis S, Surace R, Ashman O. Treatment of ulcerative colitis using fecal bacteriotherapy. J Clin Gastroenterol. 2003;37(1):42–47

3.Khan KJ, Ullman TA, Ford AC, et al. Antibiotic Therapy in Inflammatory Bowel Disease: A Systematic Review and Meta-Analysis. The American Journal of Gastroenterology

4.2 Indication to be Studied

Fecal microbiota transplantation and supplementation in patients with mild to moderate ulcerative colitis who have persistent evidence of active inflammation despite standard therapy.

4.3 General Approach for Evaluation of Treatment

We propose a prospective, randomized controlled trial whereby patients with mild to moderate ulcerative colitis will be randomly selected into one of two arms. Patients must be deemed refractory to current therapy with a 5-ASA compound, an immunomodulator such as 6-mercaptopurine or azathioprine, and/or biologic agent. Refractory patients are defined as those with persistent, classical symptoms of ulcerative colitis with evidence of macroscopic and microscopic inflammation on endoscopic evaluation. Those recruited into the study will continue their current medication regimens without alteration. Those graded higher than "moderate" ulcerative colitis or on steroid dependent therapy will be excluded from enrollment.

4.4 Collection of Pilot Tissue and Control Tissue and Peripheral Blood

An important aspect of this study is to elucidate the underlying physiologic mechanisms responsible for FMT's effect. Previous data suggests that FMT may effect host immune response. In order to investigate changes in host immune function associated with FMT, this study seeks to characterize changes in T-cell population phenotype at both the mucosal level as well as in the peripheral blood of study participants over time. In order to do this, mucosal biopsies and peripheral blood samples will be collected from FMT study participants at the procedure visit and at the 12 week follow-up visit. The mucosal biopsies for FMT study participants will be obtained from below the peritoneal reflection via colonoscopy and sigmoidoscopy respectively. Cell suspensions made from the mucosal biopsies will be subjected to flow cytometric analysis at the Flow Cytometry Core Lab at UVM and possible T cell receptor sequencing in collaboration with other laboratories. Only de-identified samples will be shared with collaborators with appropriate data sharing agreements in place. This process is not routine and requires procedure optimization prior to the collection of patient samples, which is why we will be collecting tissue samples from patients undergoing colonoscopy with tissue collection (*see pilot tissue collection below*).

Pilot Tissue Collection

The optimization process for Flow Cytometry and RNA analysis must be done with tissue as similar as possible to those that will be collected within the FMT study. For this purpose, tissue will be obtained from two sources; 1) Ulcerative Colitis (UC) patients undergoing routine screening colonoscopy and 2) surgical tissue obtained as part of routine patient care. The study team anticipates the collection of pilot tissue from 7-10 individuals for this purpose. This optimization process will be completed prior to processing tissue samples from study patients.

Pilot tissue will also be collected in order to optimize tissue processing for T cell receptor sequencing. This process involves staining and sorting tissue-based mononuclear cells with subsequent RNA isolation at our collaborator's core facility at MIT. Pilot tissue shared with our collaborators for this purpose will be deidentifed. A material transfer agreement is in place for this purpose..

Mucosal biopsies from UC patients undergoing routine screening colonoscopy will be collected through a separate consent process for this purpose. Three mucosal pinch biopsies will be taken for this purpose from 7-10 patients undergoing colonoscopy for clinical care who are already having biopsies taken. All pilot tissue will be taken from below the peritoneal reflection in order to limit risk to the patient (possible perforation).

Alternatively, a portion of surgical tissue obtained in the process of routine clinical care (i.e. colectomy specimens for cancer removal) can be used by the research team in order to technically validate research protocols involved with this study. The use of this tissue will not alter patient care in any way. Only non-diagnostic tissue (ie tissue spatially removed from pathologic findings, such as tumors and margins) will be used. This tissue will be designated by a pathologist or their appointee (ie Pathologist Assistant), and not chosen by the research team. No identifying information will be obtained in association with this tissue and thus there is no anticipated risk to the source patient's confidentiality.

Additionally, the resultant histologic slides of clinically-obtained tissues (biopsies and surgical specimens) obtained as part of the patient's routine medical care (for cancer screening or another medical indication) may be assessed by the research team *after* they have been assessed by a pathologist and used for diagnosis in a medical setting.

Control Tissue and Peripheral Blood Collection

This study seeks to characterize the mucosal T-cell phenotype of study patients. Mucosal immunity is extremely complex and little is established in the literature regarding normal baseline mucosal immunity over time. In order for this study to reach meaningful conclusions, a baseline point of comparison must be established. Biopsies and approximately 20 mLs of peripheral blood from normal non-Ulcerative Colitis patients undergoing routine screening colonoscopy will be collected through a separate consent process. The biopsies and blood samples will be processed in an identical manner as those from study patients in order to establish this baseline. In order to minimize risk, the biopsies will only be collected from below the retroperitoneal reflection. The study team anticipates the collection of control biopsies and blood samples from 5 individuals for this purpose.

Additionally, the resultant histologic slides of the biopsies obtained as part of the patient's routine medical care (for cancer screening or another medical indication) may be assessed by the research team *after* they have been assessed by a pathologist and used for diagnosis in a medical setting.

Interim Analysis

The original recruitment goal of 20 subjects was not based on a sample-size calculation. The PI, with the agreement of all co-investigators has decided to hold enrollment and conduct an interim data analysis using the 12 subjects who have been randomized thus far. The data analyzed will include: clinical information, 16S microbial DNA sequencing, Short-Chain Fatty Acid content in stool samples, serotonin signaling form biopsies, histopathology, and flow cytometry. In order to fully understand the relevance of FMT in UC patients all clinically blinded personal that were blinded during the enrollment period will remain blinded during the interim analysis period. Biostatistical support will be limited to those that have been included in the HIPAA authorization and have a legally binding contract with UVMMC. Final analysis will be conducted at the University of Vermont, The University of Vermont Medical Center, and OpenBiome (Somerville MA).

4.5 Description of First Year Trials

Patients will be enrolled into the study based on their global assessment as mild or moderate active ulcerative colitis that is refractory to current treatment with a 5-ASA compound, an immunomodulator, and/or a biologic. Any patient with "severe" colitis will be excluded from the study, as will any patient with recent antibiotic use (<30 days), current steroid therapy, or active infection of any kind. Further exclusion criteria involve patients diagnosed with Crohn's disease, and patients with a surgical history for IBD exclusively.

Patients will be randomized into two separate cohorts:

1. Sham FMT by colonoscopy at induction and sham oral capsules administered daily for 12 weeks

2. FMT by colonoscopy at induction and oral FMT capsules administered daily for 12 weeks

At the time of randomization, all subjects will complete the subjective MAYO assessment, IBDQ and SF36 questionnaires—validated measures to track IBD symptom severity and quality of life. They will also provide blood and stool samples for standard serologic (CRP) and fecal (calprotectin and lactoferrin) inflammatory markers. Stool samples will also be collected at baseline for metagenomic sequencing in order to characterize each patient's enteric microbiome. Patients in each cohort will then take a 7-day course of ciprofloxacin 250mg q12 hours combined with metronidazole 500mg q8 hours (standard dosing) and then undergo a baseline endoscopic evaluation via colonoscopy within 48 hours of antibiotic completion. Each subject will be evaluated according to an endoscopic MAYO and Ulcerative Colitis Endoscopic Index of Severity (UCEIS) score. Random biopsies from the sigmoid colon and rectum will be collected and interpreted by two independent pathologists who are blinded to clinical data for histologic grading (quiescent/mild/moderate/severe disease activity). Additional biopsy specimens will also be collected for research purposes only. (See section on Investigation of Mechanism below). A blood samples will be collected at this procedure visit to measure inflammatory markers (CRP) and for research purposes (See section on Investigation of Mechanism below).

Upon completion of baseline endoscopic evaluation and collection of mucosal biopsies, induction therapy will commence. Patients will receive FMT vs sham treatment depending on the cohort to which they have been randomly assigned. After induction therapy, patients will bring home a 4 week supply of oral capsules. The capsules will be stored with an icepack and a small amount of dry ice in a Styrofoam cooler and kept in patient's home freezer. Dry ice will be placed in the cooler in order to mediate temperature changes during the transportation of the capsule from the clinic to the patient's home freezer. An information sheet surround the proper handling and disposing of dry ice will be provided to the patients. Patients will come to the clinic at the commencement of 4 weeks of therapy to pick up their second 4 week supply of oral therapy. At the end of the second 4-weeks of therapy (week 8 visit), patients will come into the clinic to pick-up their final 4-weeks of therapy and have a blood draw only. In total, patients will take 1 capsule oral daily microbiota vs placebo capsules for 12 weeks, according to their assigned cohort.

All subjects will complete additional subjective MAYO, IBDQ and SF36 questionnaires at their 4 week, 12 week and 18 week visits. Repeat blood samples will be collected to measure CRP at each of these visits. Stool samples will be collected at each of these visits to measure fecal lactoferrrin and patients will collect at-home stool samples on a weekly basis after the screening visit for research purposes. A flexible sigmoidoscopy will be performed on an outpatient basis within 48 hours of FMT completion for the recording of a post-treatment endoscopic MAYO and UCEIS scores performed by a blinded endoscopist. Biopsies will be sampled from the sigmoid colon and rectum and interpreted by two independent pathologists who are blinded to clinical data. Biopsy specimens will also be used to assess changes in serotonin signaling and the immunophenotypic profile of patient's mucosal inflammatory response after FMT. Peripheral blood samples will be obtained at the flexible sigmoidoscopy visit to study changes in immune response and serum metabolites.

4.6 Number of Subjects to be Evaluated

A total of 20 patients will be randomized into the study. Ten patients will be randomly assigned to the control group and ten patients will be randomly assigned to the experimental group. The subject accrual number has been increase to 40 to account for screen failures, control and pilot subjects.

4.7 Drug Related Risks

Colonoscopy is a safe procedure associated with a complication rate in the range of approximately 1 in 1,000. The most severe, but least common complications include infection, hemorrhage, perforation, or hypoxia and/or arrhythmia related to the sedation given during the procedure. Since conscious sedation is administered, most people will experience very little discomfort, approximating a digital rectal exam. The procedure can be stopped at any time if the individual's comfort is compromised. The adverse risks of

FMT are not completely known but may include the risk of transmitting infections such as bacteria, parasites or viruses. As such, donors are screened aggressively for previous exposure to and active infections.

4.8 References

20. Rubin DT. The Emerging Role of the Microbiome in the Pathogenesis and Management of Inflammatory Bowel Disease. In: ACG 2013 Annual Scientific Meeting & Postgraduate Course; 2013:1-19.

5. INVESTIGATOR BROCHURE

The study in question will be performed at a single clinical site, and therefore an investigator brochure will not need to be provided.

5.1 Drug Substance and Formulation

Fecal Microbiota Preparation

Human Stool (active ingredient); Normal non-bacteriostatic saline (0.9%; excipient); USP Glycerol (.99% glycerol in 1% water, excipient); Cocoa Butter (excipient); Gelatin Capsules (excipient)

5.2 Pharmacological and Toxicological Effects

Fecal microbiota transplantation and stool preparation developed from empirical clinical practice instead of by way of a conventional drug development program. Accordingly, salient non-clinical data is not particularly informative as each individual's microbiome has co-evolved with its host since birth.

5.3 Safety and Effectiveness of the Drug

The safety profile of human stool for transplantation purposes is still under investigation. Adverse events related directly to fecal microbiota transplantation are rare. The added components in the fecal microbiota preparation supplied by OpenBiome are generally recognized as safe by the FDA.

The overall effectiveness of stool transplantation for various disease states is also under investigation. Current literature supports the use of fecal transplantation in recurrent *Clostridium difficile* related colitis, with an effective cure rate of close to 90%. Results and adverse events in immunocompromised patients (HIV/AIDS, solid organ transplantation, oncologic conditions, immunosuppressive therapy for IBD, or due to medical condition/medication) have been shown to be similar to those of immune-competent patients. For the purposes of this study all immunocompromised patients will be excluded from participating.

5.4 Risks and Side Effects

The potential risks of fecal microbiota transplantation are not completely known but may include the risk of transmitting infections such as bacteria, parasites or viruses. Fecal microbiota transplantation in theory may be associated with the development of autoimmune diseases such as rheumatoid arthritis, Sjogrens syndrome and peripheral neuropathy. Some patients have experienced an IBD flare post-FMT; however it is unclear if the flare of activity is related to the actual transplant, as most patients do not experience such adverse events.

5.4.1 Potential risks to subjects

There are three potential areas of risk to subjects associated with participation in the proposed study

These include:

1) Physical risks related to the colonoscopy
2) Theoretical risks of FMT
3) Psychological or other risks related to confidentiality and loss of privacy

Risks of ingestion of the colon preparation include the risk of dehydration and minor electrolyte imbalances. Standard colonoscopy risks include the risk of bowel perforation, bleeding, and adverse cardiopulmonary events related to sedation. The infusion of the liquid fecal matter will prolong colonoscopy by less than five minutes and adds no additional risk to the colonoscopy. Many adverse effects of colonoscopy resolve shortly after the procedure has been completed, but in some cases abdominal discomfort and gaseous side pain can persist for several hours.

There have been no confirmed infectious complications directly attributable to FMT reported in the literature aside from a speculation about a norovirus case. There have been two FMT cases associated with bacteremia, although the strength of the association is unclear and both patients did well on antibiotic therapy. Since this process involves infusion of a biological agent from one person to another, there remains a theoretical possibility of transmitting an infectious agent. Risks will be minimized by a rigorous donor selection process that includes an evaluation of both stool and blood for common, yet dangerous infections agents. Subjects will be informed that the treatment or procedure may involve these theoretical risks and additional risks that are currently unforeseeable. As mentioned previously, there have been reported cases of inflammatory bowel disease flares following FMT cases, but no correlation has been established. Infusion of fecal material containing a potential agent to which the subject is allergic is also a concern. To minimize this risk, patients with a history of severe (anaphylaxis) allergy will be excluded from this study.

5.4.2 Potential Risks to Donors

This study also involves donors recruited by OpenBiome to donate stool for FMT. There are three areas of risk to the potential stool donor
These include:

1) Physical risks related to laboratory testing and stool collection protocol
2) Psychological risks related to revealing sensitive information during the screening process
3) Risks related to confidentiality and loss of privacy

OpenBiome volunteer donors will be utilized. In order to exclude donors at high risk of passing infection, a Donor Risk Factor Questionnaire will be administered. A sample of this questionnaire is included with the submission of the drug master file. The questionnaire contains potentially sensitive information, including any history of incarceration, drug use, and high-risk sexual behaviors. Additionally, prospective donors will be asked questions about their baseline health status and co-morbidities. Laboratory tests drawn as part of the screening process will include testing for HIV, viral hepatitis, and syphilis. These serologic results, if found to be abnormal, could cause psychological distress to the donor. Potential donors may experience psychological distress if they are excluded from donating stool based on these screenings.

5.4.3 Protection against Risks to Subjects and Donors

Participants will be recruited from the study site as described in the research protocol if they initially meet all of the inclusion and exclusion criteria. The research staff will carefully explain all aspects of the study to a potential recruit, including the risks and benefits prior to obtaining consent.

- The research assistant or clinician will orally describe the material written in the informed consent document and answer any questions the participant may have.
- Participants will be reminded that they are not required to participate in the study and that they will receive the standard of care provided by their physician regardless of such decision. Participants who give their consent will sign a copy of the document and will be given a signed copy of the informed consent document.

- Proper documentation of the informed consent process will be completed for all subjects that chose to participate in the study. A copy of the signed informed consent form and HIPAA will be provided to the subject.

Recruits will be informed of the treatment commitment, including the amount, and general types of assessments, the follow-up telephone interview procedures and clinic visits. They will be given detailed descriptions of the colonoscopy with FMT procedure. They will be informed of the protocol with respect to collecting blood and stool samples at intervals throughout the study period.

Potential donors who are healthy will be recruited by OpenBiome and contacted by OpenBiome directly to participate in the donor consent process. Research staff will carefully explain the aspects of the study to a potential donor, including the risks and benefits, and obtain participants' written informed consent. Recruits will be informed of the commitment including a detailed description of the donor screening process and a stool donation protocol. The research staff will explain that a thorough clinical assessment and screening questionnaire will ask potentially sensitive questions regarding any history of incarceration, drug use, and sexual habits. They will be informed of the requirements to have laboratory blood work, including HIV testing, and stool studies. The research assistant will orally describe the material written in the informed consent document and any questions the potential donor may have. Potential donors will be reminded that they are not required to participate in the study. Participants who give their consent will sign a copy of the document and will be given a signed copy of the informed consent document.

Data and safety monitoring will take place to assure the safety of subjects. All participants will be reminded that their participation is voluntary and that they can withdraw from the study at any time without penalty. Risk will be minimized by the following procedures:

1) We will minimize the risk of potential coercion by following standard procedures for obtaining informed consent from both subjects and stool donors. We will begin this process during the intake, where we will clarify the nature of the study and possible alternatives upfront. Prior to enrolling subjects in the research, we will fully explain the study procedures, risks, benefits, and alternatives, emphasizing that the subject's participation has no impact on the other medical services they receive. Also, subjects who do not consent or who withdraw during the study period will continue to receive appropriate treatment if needed. All subjects and donors will be reminded that there is no penalty for those who choose not to participate or to withdraw from the study and that their decision to participate does not affect their access to services provided by the medical institution. Subjects will not receive financial compensation for their participation. Volunteer donors will be paid a small stipend of $40 by OpenBiome per stool donation.
2) We will minimize potential risks due to loss of confidentiality by having all information collected and handled by research staff trained to deal appropriately with sensitive clinical issues. Potential donors will also be informed about the risk of being ineligible to donate stool due to positive results on screening questionnaires or laboratory testing. Results of donor medical interview, screening and laboratory testing will be kept separate from subject data and only de-identified data will be available to the subject or his/her most responsible physician. All study information will be treated as confidential and will be available only to research staff. Hardcopies will be kept in locked file cabinets, and computer data files will be encrypted and available only to authorized personnel- with no storage of names or obvious identifying information. No participant will be identified in any report of the project. Further, when contacting participants for follow-up, no identifying information other than the first name of the research assistant will be used when leaving messages or speaking to anyone other than the participant him/herself. Written consent will be obtained to contact other persons for the purpose of locating the participant for

follow-up and participants can refuse or revoke such consents. No information about participants will be released without their permission or where required by law.

3) We will minimize the theoretical risk of infectious disease or other conditions possibly transmitted through FMT by using stool obtained via donor screening protocols modeled after blood banks and organ transplant programs. Potential volunteer donors will undergo thorough screening to determine eligibility for donation. The primary purpose of the donor examination and interview is to ensure that the donor is in good health, and to identify risk factors for diseases transmissible by stool. The donor interview will be used to identify risks for diseases and conditions for which there are no laboratory tests, for which tests are not sensitive enough to detect infectious agents, and for tests unable to identify early stage or window period infections. Potential donors will be interviewed using a Donor Risk Factor Questionnaire. Health status of donors will be monitored prospectively, and donors will be rescreened every 60 days.
4) We will minimize the risk of a potential exacerbation of ulcerative colitis by maintaining close clinical contact with all subjects. Subjects will be encouraged to contact the clinical team if they experience worsening diarrhea, hematochezia, fever, abdominal pain, nausea, or further signs of decompensation. We plan to call all patients 24 hours after their initial colonoscopy, regardless of treatment arm to assess for any complications or adverse events following the procedure. In addition, all patients will be evaluated by at least one member of the primary research team within 24 hours following a serious adverse event. If indeed a patient does have a flare of symptoms, standard of care will be delivered to induce remission.
5) We will minimize the potential risk to a fetus with reproductive counseling for all women of child bearing age at the initial screening visit and by requiring the use of contraception throughout the study period. The study team will also perform urine pregnancy tests at clinic visits for women of child bearing potential.

5.4.4 Safety Monitoring Protocol

The Principal Investigator will review all AEs and new safety information in real time and monthly. The PI will practice standard procedures for monitoring and reporting. The PI will evaluate recruitment, the progress of the study, subject retention, data quality and confidentiality. The PI will review subjects’ clinical status, adverse events and whether or not there have been any changes in risk to participating subjects. This monthly review will ensure that subject risk does not outweigh study benefits. Any new safety information or any AEs in relation to FMT will be forwarded to the IRBs and to the FDA (in accordance with Investigational New Drug (IND) regulations).

The safety of participants will be monitored for the duration of the study at each contact. Both anticipated and unanticipated adverse events and problems will be formally monitored and recorded. Unanticipated serious adverse events related to FMT material will be reported to the hospital and IRBs (as per local reporting requirements) and the FDA (within 15 days; or 7 days for unexpected fatal or life threatening events related to FMT material). Anticipated less serious adverse events, as previously outlined will be submitted annually in reports to the IRBs and FDA. The Principal Investigators will be responsible for monitoring the safety and efficacy of the study, and complying with reporting requirements. All AEs will be recorded and properly documented and the severity will be rated (mild, moderate, severe) for every subject and will be properly reported per GCP guidelines to the local IRB and the FDA. All subjects will be advised to call the PI for any AE that they may experience (e.g. fever, headaches, ER visits) as soon as possible. Subjects will also be made aware of the importance of reporting AEs, and non-compliance may increase their risk. 24-hour emergency contact information will be provided to

all subjects at the time of consent, this will allows us to properly access and document all adverse events.

Data will be collected using standardized paper forms and will only be identified with the study's ID of the subject. The codes that link the name of the subject and the study ID will be kept confidential by the Site Investigator in a secured cabinet. Collected forms will be transported to the Site Investigator data entry center.

The following definitions will be used by the primary investigator to assess causal relationship/relatedness between the investigational product and an adverse event.
- **Unrelated** to study product (the AE clearly NOT related to the intervention)
- **Unlikely** related to the study product (the AE is doubtfully related to the intervention)
- **Possibly** related to study product (the AE maybe related to the intervention)
- **Probably** related to study product (the AE is likely related to the intervention)
- **Definitely** related to study product (The AE is clearly related to the intervention)

In order to enhance safety to study participants, individual halting rules will be as follows:

a. A serious adverse event (SAE) that is judged to be at least possibly related to the study product
b. Any grade of hypersensitivity reaction to the study product
c. Pregnancy during the period of study product administration

In addition, study halting rules will be implemented and are as follows:

a. The occurrence of an SAE that is judged not unrelated to study product administration
b. The occurrence of a potentially fecaly-transmitted infectious disease
c. The occurrence of 3 or more similar grade 3 adverse events

5.4.5. Data Monitoring Plan

Data will be collected using standardized paper forms and will only be identified with the study's ID of the subject. The codes that link the name of the subject and the study ID will be kept confidential by the Site Investigator in a secured cabinet. Collected forms will be transported to the Site Investigator data entry center. Data will be entered in the REDCap computer database independently by trained data entry staff, and discrepancies will be corrected by the Site Investigator, based on source documents. Data quality will be monitored once per month by random inspection of the completed forms by a Clinical Research Coordinator; any problems detected will be discussed with the Principle Investigator. Telephone follow-up will be conducted by a Clinical Research Coordinator with data directly entered immediately into the REDCap computer database. Descriptive statistics will be computed periodically and reported in aggregate as part of the study monitoring process.

5.4.6. Educational Training

Researchers and clinician Investigators involved in the study will need to have successfully completed an institutional human subjects research program/module. The principle Investigator will maintain documentation for all study personnel. The research team will so have signed a delegation of authority log; this log will be properly

maintained and updated with new staff. All staff will also sign a training log regarding protocol and amendment training.

6. PROTOCOL

6.1 Study protocol

Ulcerative colitis patients 18-75 years of age with mild to moderate active disease on standard medical therapy will be recruited. These patients are felt to represent a “middle of the road” patient population and are hypothesized to benefit most from FMT therapy. Individuals with severe disease who are refractory to medical therapy will be avoided, as prior trials have shown less promising results in this group and it is anticipated that more aggressive therapy may be indicated. Completely asymptomatic patients will likewise be excluded as they do not stand to derive benefit and should thus not incur potential risks associated with FMT, however hypothetical. The following list of inclusion and exclusion criteria will be used. Generally, these criteria are aimed at limiting variability within the study cohort in order to minimize confounding variables and standardize patient characteristics.

Patients may be on any IBD-related medication with the exception of steroids (systemic or topical) prior to enrollment, provided that they are on stable dosages for at least 6 weeks. This is important in order to limit treatment variability and thus strengthen insight into potential FMT treatment effect.

If clinically indicated patients will be tested for *Clostridium difficile* (by PCR) or other enteric pathogens by stool culture and if positive will be excluded in order to limit confounding microbiologic variables. Prior systemic antibiotic use within 6 weeks and/or probiotic use within 2 days will also be avoided for this reason.

At risk populations, including those with severe immunodeficiency and pregnant women, will be avoided due to potential risks of infection. Urine pregnancy tests will be completed at each scheduled visit if the subject is female and is of child bearing age. Patients will be counseled to avoid pregnancy for the duration of the study. In the event of an unplanned pregnancy, subjects will be asked to cease all study medications and supplements. They will not be included in study analyses, however will be followed by our medical team in conjunction with their primary care/obstetrician for the duration of the pregnancy.

Patients will be randomized by an unblinded pharmacist to 1 of 2 arms with no crossover. All other study personal will be blinded.

Measurement of IBD severity by symptoms

At time of study enrollment, at 4 weeks, upon completion of the FMT protocol at 12 weeks, and at 18 week follow-up, subjects will be asked to complete the MAYO symptom score, IBDQ and SF36 questionnaires—validated measures to track IBD symptom severity and quality of life. At 4 weeks, all subjects will be seen in clinic for personal evaluation.

The physician or study coordinator will provide the MAYO, IBDQ and SF-36 survey to the patient directly. The patient will be provided a private area to complete the questions. The documents will be coded with a de-identified patient ID number. These surveys will be scored and stored in a locked computer in a locked room. The only people with access to the room and computer are the study coordinator and investigators.

Measurement of IBD severity by histology

At time of induction colonoscopy, pre-treatment biopsies will be taken to measure disease activity by histology (defined as quiescent, mild, moderate, or severe). Upon completion of FMT at 12 weeks, subjects will undergo an outpatient flexible sigmoidoscopy. At this time post-treatment biopsies will be taken for comparison of histologic disease activity. Two independent pathologists will confirm histologic grading. When disagreement occurs, a third pathologist will independently evaluate the sample and all three pathologists will come to a consensus.

Measurement of IBD severity by serologic and fecal inflammatory markers

Prior to, during and upon completion of the FMT protocol all patients will provide blood and stool samples for standard serologic (CRP) and fecal (calprotectin and lactoferrin) inflammatory markers. These markers are frequently used in clinical practice and are indicated to characterize disease activity in symptomatic patients.

Measurement of Diet Impact on the Gut Microbiome

The diversity of plants in an individual's diet is associated with changes in their gut microbiome. In order to capture this information, patients will be asked to record the number of different plant species they consume each day in a daily log. The plant species log will be provided to the subjects and will be asked to fill it out daily during the study period and will be collected at the week 18 visit.

Investigation of Mechanism

Tissue biopsies procured pre and post FMT (or placebo) treatment will be placed in RPMI and brought to our core research facility (UVM-Flow Cytometry and Cell Sorting Facility) by study team members. Cell suspensions will be made and incubated overnight for cytokine secretion. Flow cytometric techniques will then be used to assess membrane markers and intracellular cytokine present in isolated lymphocytes populations. The purpose of which is to identify and quantitate changes in effector lymphocyte populations. Procedures will be optimized prior to processing study patient samples, please see section on the collection of pilot tissue.

Peripheral blood will be collected from study participants pre and post FMT (or placebo) treatment. These samples will be processed in our core research facility (UVM-Flow Cytometry and Cell Sorting Facility) by study team members. The cellular component of these samples will separated from serum. Flow cytometric techniques will be used to identify and quantitate circulating populations of effector lymphocyte populations within the cellular component.

Peripheral blood will also be collected at weeks 0, 4, 8 and 12 in order to study changes in T-cell response by molecular technologies including Polymerase Chain Reaction (PCR) and sequencing-based techniques such as Human Leukocyte Antigen (HLA) typing, and serum metabolomics by mass spectometry. These samples will be de-identified, coded and then processed at a collaborating lab at MIT. A usage agreement has been executed for this purpose.

Tissue biopsies procured pre and post FMT (or placebo) treatment will be placed in RNA later and brought to a study team member's lab at UVM where qRT-PCR techniques will be used to assess for changes serotonin signaling molecules.

Statistical Considerations:

The outcomes being measured are listed in above sections. This is a pilot study with a goal recruitment of 20 patients, 10 in each arm. This study is not powered to show statistically significant treatment effect. This study is

instead designed to look for trends and gauge the utility of this novel protocol in this patient population. Additionally, this study will offer preliminary data related to potential FMT mechanism of action.

Confidentiality Measures and Secure Storage of Data or Tissue:

Clinical information specifically related to this study as well as prospectively collected research data (including endoscopic reports) will be stored in electronic databases maintained on password protected computers kept in locked offices. Any hardcopy data will be kept in offices/laboratories that are locked when unoccupied. Data will be accessible to the project team only.

Tissue biopsies obtained at initial colonscopy and follow-up flexible sigmoidoscopy will be processed and maintained by standard protocols within the pathology department of UVMMC. Any slides derived from these biopsies solely for research purposes will be maintained in a de-identified manner by the research team within a locked office in the UVM pathology department.

Blood, tissue and stool samples obtained solely for research purposes will be labeled with a research number and date; no identifiable information will be recorded on these samples. Samples will be maintained by the research team within the UVM pathology department.

Stool samples collected by the patients for microbial metagenomic sequencing will be labeled with a research number and date only and sent directly to our collaborators, OpenBiome, for storage in their research facility until sequencing. No identifiable information will be shared with non-UVMMC research collaborators.

Sources of Material:

The following samples will be obtained in a de-identifed manner and will not be traceable to individual subjects:
- Stool Samples for metagenomic sequencing
- Blood samples for serum metabolites, PCR and flow cytometery analysis
- Mucosal Biopsy Samples for immunophenotyping and measuring serotonin signaling.

The following samples will be obtained in an identifiable manner and results will be the subject's medical record:
- Fecal samples for fecal lactoferrin and fecal calprotectin
- Blood samples for serologic markers of inflammation (CRP)

Therapeutic Alternatives:

Therapeutic alternatives for patients with mild-moderate UC will depend on the patient's comorbidities and current IBD medical regimen. This will be discussed with each patient and is clearly delineated in the consent form.

Subject Characteristics, Identification and Recruitment

Minorities and women will be included in the study. Children will be excluded due to pediatric IBD management variability. In an effort to minimize cohort variation in this small, pilot study adults defined as greater than or equal to 18 years of age will be included. HIV testing will not be necessary.

Recruitment will be used. Dr. Peter Moses, the primary investigator will write an advertisement for local newspapers. Local newspapers and media will be contacted regarding a potential article or story surrounding the study. The information that is provided to these news outlets will be the same as that found on the

clinicaltrials.gov website. Prior to the article being made public the team will require final approval. There will also be an advertisement that will run on Front Porch Forum that will direct potential subjects to the University of Vermont contact page. This will then allow the subject to fill out their contact information so the study team can contact them. The Facebook, Twitter, and Google + accounts of the University of Vermont Medical Center and OpenBiome will also be used for advertisement and recruitment for this study. If a subject is interested they can click on a social media links that will allow a potential subject to provide personal information to the research team in order for the team to contact them about the study. Peter Moses will also post a blog about this study on The University of Vermont Medical Center website, there will also be a link on this website that will also patient to enter contact information that will be sent to the research team. Advertisements will be posted on The University of Vermont Medical Center website. Peter Moses will send out a letter to all gastrointestinal providers in Vermont and New York informing them about this clinical trial. The recruitment material is attached for reference. The study will also utilize a flyer containing information about the study and the contact information, this flyer is attached for your review.

Subjects will not be paid to participate in this study. All study medications (expect the colo-prep GoLytely) will be provided to the patients by internal funding from UVMMC. Subjects will not incur any charges through participation in this study apart from the colonoscopy procedure, resultant biopsies, and the GoLytely prep medication. As the flexible sigmoidoscopy will be performed for research purposes, it and its resultant biopsies will not be billed to the patient and will be cover by research study funds.

Inclusion Criteria:

- Men or women 18-75 years of age.
- Established diagnosis of ulcerative colitis (UC) with known involvement of the left colon
- Mild to moderate disease defined as endoscopic evidence of disease with Mayo endoscopic sub-score 1 or 2 and total MAYO score ranging from 4-10. (The Mayo score ranges from 0 to 12, with higher scores indicating more severe disease. This score can be used for both initial evaluation and monitoring treatment response).
- Patients may be on any class of IBD-related medication (excluding steroids)
- Patients must be on stable medication regimen for at least 6 weeks prior to enrollment.
- Ability to understand and willingness to sign informed consent document

Exclusion Criteria:

- Patient who are asymptomatic
- Patients with severe, refractory disease (defined as Mayo scores of > 10, or endoscopic disease activity score of > 3) or patients with any other significant condition which, in the opinion of the investigator, could confound or interfere with evaluation of safety, tolerability of the investigational treatment or prevent compliance with the study protocol
- Prior colectomy
- Positive stool test for any of the following: *Clostridium difficile* by PCR, *Salmonella, Shigella, Yersinia, Campylobacter, enteropathogenic E. coli* by standard stool culture.
- Use of the steroid medications (any formulation, excluding inhaled steroids) in the prior 6 weeks to enrollment
- Systemic antibiotic use within prior 6 weeks to enrollment
- Probiotic supplement use within 2 days prior to enrollment
- Pregnancy or breastfeeding
- Severe immunodeficiency, inherited or acquired (e.g. HIV, chemotherapy, or radiation therapy)
- History of anaphylaxis (severe allergic reaction)
- Documented allergy to fluoroquinolones, metronidazole
- Life expectancy less than 12 months
- Age less than 18 or greater than 75 years of age

- History of esophageal or gastric motility disorders.

Collaborating Sites:

This research will occur in partnership with OpenBiome, a non-profit stool bank based in Medford, MA. OpenBiome has a Biologics Drug Master File registered with the FDA (BB-MF 15543) and can be referenced at fda.gov. OpenBiome's FMT product is currently in use in healthcare institutions across the country, including the University of Vermont Medical Center.

OpenBiome will provide the fecal material for transplantation, both for colonoscopic infusion and in oral capsule formation. The stool is donated by healthy monitored individuals, screened for infections, and prepared to a proper consistency in accordance to OpenBiome's standard procedures. Please see accompanying organization protocols.

OpenBiome in collaboration with MIT will also aid in genomic stool analysis by both 16S ribosomal and metagenomic sequencing of patient stool samples. Stool samples will be collected by patients at home into specially formulated stool collection kits. Kits will be mailed via United States Postal Service directly by the subject to OpenBiome/MIT. Kits will be labeled with study number and date and time of sample collection only; no patient names or identifying information will be included on the kits. Once received, the samples will be maintained in a research freezer maintained by OpenBiome at minus 80 degrees Celsius until sequencing facilitated by MIT.

OpenBiome in collaboration with MIT will also aid in the processing of de-identified blood samples obtained for research purposes. Samples will be coded and no personal health information will be shared. A usage agreement has been executed for this purpose.

Informed Consent:

Consent for participation in this study will be obtained by the Primary Investigator or a Co-investigator within a private health care setting. The patient will be given information regarding FMT, alternative therapies, the risks and benefits associated with FMT and the fact that this is a randomized control trial meaning that participation may result in administration of a placebo and they may not actually receive FMT. The patient will be given ample time to ask questions. If the patient chooses to participate, they will sign the designated consent form, which will be maintained by the research team in a locked office.

No information will be withheld from subjects.

Screening Visit:

- Signing of informed consent and HIPAA (per GCP guidelines)
- Assessment of inclusion/exclusion criteria with the subject
- Questionnaires (Mayo symptom score, IBDQ and SF-36)*
- Dispense Plant Species Log
- Stool sample to measure inflammatory markers fecal lactoferrin and fecal calprotectin

- Women of child bearing age will be counseled regarding pregnancy and the need for contraception for the duration of the study. A urine pregnancy test will be performed on all women with child bearing potential.
- Blood collection: Baseline serologic inflammatory markers (CRP)
- Baseline stool sample: Patient will collect 3 baseline stool sample at home and send to research team by US Postal Service for storage until sequencing.
- Patients in both the control and experiment arm of the study will be pretreated with antibiotics for 7 days (ciprofloxacin 250mg PO q12 and metronidazole 500mg PO q8 x7 days)

Procedure Visit/Induction Colonoscopy:

- Prior to the colonoscopy subjects will consume 4 liters of polyethylene glycol (2 liter the night before, 2 liter the morning of) at a rate of 8oz every 10 minutes.
- Colonoscopy
- Record Endoscopic MAYO and UCEIS score
- Blood draw for research purposes (circulating T-cell populations by flow cytometry* and PCR, and serum metabolites via mass spectrometry).
- Rectal biopsies to assess histopathologic disease burden, serotonin signaling and immunophenotypic profile of mucosal immune response.
- FMT or sham FMT administered by blinded endoscopist*.
- Patients will leave endoscopy suite with a 4-week supply of FMT (or sham FMT) capsules inside a Styrofoam cooler with an icepack and small amount of dry ice and a medication adherence log.
- Patients will be given a 4 week supply of at home weekly stool collection kits.
- Patients randomized to either control arm or experimental arm.

4 -Week Visit:

- Outpatient visit at 4 weeks upon completion of FMT or Placebo capsules
- Repeat Mayo symptom score, IBDQ and SF-36 questionnaires*.
- A urine pregnancy test will be completed if applicable.
- Stool sample to measure fecal lactoferrin
- Blood draw to repeat inflammatory mediators (CRP)
- Blood draw for research purposes (T cells by PCR and metabolomics)
- Adverse events, concomitant medications, and medical history will be reviewed.
- Patients will leave endoscopy suite with second 4-week supply of FMT (or sham FMT) capsules inside a Styrofoam cooler with an icepack and small amount of dry ice and a medication adherence log.
- Dispense Plant Species Log
-
- Patients will be given a 4 week supply of at home weekly stool collection kits.

8- Week Visit:

- Outpatient visit with study coordinator upon completion of second 4-week batch of FMT or Placebo capsules- Blood draw for research purposes (T cells by PCR and metabolomics)
- Patients will leave visit with third 4-week supply of FMT (or sham) FMT capsules inside a Styrofoam cooler with an icepack and small amount of dry ice and a medication adherence log.
- Patients will be given a 4 week supple of at home weekly stool collection kits.
- Dispense Plant Species Log

12 -Week Visit:

- Outpatient visit at 12 weeks upon completion of FMT or Placebo capsules
- Repeat Mayo symptom score, IBDQ and SF-36 questionnaires*.
- A urine pregnancy test will be completed if applicable.
- Flexible sigmoidoscopy to record endoscopic MAYO and UCEIS scores and collect rectal biopsies to assess histopathologic disease burden, serotonin signaling and immune-phenotypic profile of mucosal immune response.
- Stool sample to measure fecal lactoferrin and fecal calprotectin
- Blood draw to repeat inflammatory mediators (CRP)
- Blood draw for research purposes (T cells by PCR and metabolomics, measure circulating T-cell populations by flow cytometry*, and assess changes in serum metabolic profile via mass spectrometry).
- Dispense Plant Species Log
- Adverse events, concomitant medications, and medical history will be reviewed.

18 Week Visit:

- Outpatient visit at 18 weeks as follow- up
- Repeat Mayo symptom score, IBDQ and SF-36 questionnaires*.
- A urine pregnancy test will be completed if applicable.
- Stool sample to measure fecal lactoferrin
- Blood draw to repeat inflammatory mediators (CRP)
- Adverse events, concomitant medications, and medical history will be reviewed.
- Sent home with 3 stool collection kits for follow-up metagenomic analysis.

36 Week Phone Call:

- Assess adverse events
- Review concomitant medication
- Review medical history

Traveling during Maintenance Dosing Period

In order to maintain capsule viability subjects who have previously planned travel dates will not be allowed to enroll until return. While we encourage patients NOT to travel during the 12 week maintenance, we recognize that extenuating circumstances may arise, including family and/or personal emergencies. If a subject must travel, they will be allowed to "miss" up to three daily doses. They will be instructed to ingest all of the missed doses at one time upon return. For example if they miss three days, then on the fourth day they will take four capsules (that day's capsule and the three they have missed). Subjects will be instructed to contact a member of the research team prior to emergency travel. These missed doses must be approved by the PI in advance. If they require more than three days of travel, they will be withdrawn from study participation due to lack of "maintenance dosing" that is required of this protocol.

Measuring the Microbiome over Time:

All patients will be asked to collect stool samples at home. Ideally, 3 samples will be collected at baseline prior to pretreatment with antibiotics, 1 time after antibiotic pretreatment and before induction therapy and then weekly (for 12 weeks) during their enrollment, regardless of assigned study arm and at the 18 week follow-up time point. In order to facilitate stool collection, OpenBiome/Massachusetts Institute of Technology (MIT) has provided stool collection kits containing 1 labeled, 30-ml leak proof stool collection vial with non-preservative fluid (Para-Pak Clean Vial, Catalog # 900312), a plastic dropper, 1 pair of nitrile gloves, and an IATA-approved preaddressed/prepaid mailer with absorbent square. The kits also come packaged with clear instructions and diagrams on how to obtain an adequate specimen at home. For reference purposes the instructions have been posted to the appendix of this document (see Appenix V). The collection vial will be sent to OpenBiome/MIT for 16S ribosomal sequencing. The information gathered from each specimen will be shared exclusively between the principal investigator at the University of Vermont Medical Center and OpenBiome/MIT. A usage agreement between the University of Vermont Medical Center and OpenBiome/MIT has been executed for this purpose.

Additionally, aliquots of stool from each vial will be stored in an IRB-approved (CHRMS: 14-611) biorepository at The University of Vermont Medical Center for future metagenomic analysis. See Biorepository Protocol below.

A small aliquot of fresh stool from the clinically collected samples provided at the screening 4 and 12 week visits for inflammation markers (lactoferrin and calprotectin) will also be store in the biorepository for future research. These samples are important because, unlike the sample collected for microbiome characterization by metagenomics sequencing, they will enable active future research using the microbial populations present in Ulcerative Colitis patients. These samples will be flash frozen and capable of being used for experiments such as animal models of fecal transplant at a later date. Patients who do not consent to the biorepository portion of this protocol will not have their samples collected for this purpose. This language has been updated in the IRB-approved (CHRMS: 14-611) protocol to reflect the additional sample collection.

Biorepository Protocol

The purpose of the stool repository is to preserve samples of fecal matter from Fecal Microbiota Transplant (FMT) recipients and donors for future metagenomic research and laboratory experimentation. Metagenomics uses next generation sequencing technology to characterize complex microbial communities and the collective genes present in an environmental sample. These lab experiments include animal models of fecal transplant from a frozen sample in order to actively study microbial organisms.

In order to protect patient privacy, the collection vials will be assigned a specific barcode with a corresponding serial number which will be matched to each patient. The matching information will be securely stored at the primary clinical site (University of Vermont Medical Center) in a locked cabinet and suite.The only perceivable adverse event would be a breach in confidentiality. The patient’s privacy and confidentiality will be maintained through strict adherence to HIPAA protocols and regulations. Upon receipt of the research stool samples, they will be de-identified and given a research number. The link between this number and patient and identity will be securely maintained in a password-protected database to which only designated research team members have access. Information regarding patient health and identity will not be shared except in approved research contexts that are in keeping with the intent of the initial consent obtained from the subjects at the time of sample procurement.

The informed consent process will be initiated in the hospital or clinic in a private setting. The patient will be given ample time to ask questions. The patient will be given as much time as they need to decide whether they would like to participate. If the patient changes their mind about participating, they may opt out of participation at any time.

Patients may choose to terminate participation in this biorepository at any time. Once a written request to this effect is received by the PI, the study team will discard all repository samples and any clinical information collected that are specific to the patient and donor.

Unanticipated problems will be regularly solicited by the treatment team and PI from patients, staff and those involved indirectly with the study. If identified, these problems will be addressed by the PI and appropriate changes made to the protocol. Every effort will be made to address the underlying cause of any unanticipated problem and to protect the safety of patients. Any unanticipated problems will be reported to the IRB and affected parties.

General Information Around Maintenance Therapy:

- FMT capsules and sham FMT capsules x 12 weeks*.
- Oral 1 capsule QD therapy dispersed in three 4-week increments. Pills are stored in sealed containers within Styrofoam coolers with icepacks and dry ice for patients to keep in their home freezer. Patients to return to clinic at 4 weeks and 8 weeks to pick up and bring home second and third coolers of 4 week supplies of FMT (or sham) capsules.
- Patients will be instructed to keep the capsules inside the coolers with icepacks and dry ice in their home freezers. (Dry ice handling instructions will be provided to all subjects)
- Patients will be asked to fill out a pill log to monitor adherence of capsules.
- Plant Species Log Explained
- No planned travel

	**Screening Visit** *(Day -7)*	**Procedure Visit** *(Day 0)*	**Follow-Up Phone Call** (24hrs after FMT)	**4 Week Visit** *(Day 28+-72hrs.)*	**8 Week Visit** *(Day 56+-72hrs.)*	**12 Week Visit** *(Day 84 +-72hrs.)*	**18 Week Visit** *(Day 126 +-72hrs.)*	**36 Week Phone Call** *(Day 264 +-72hrs.)*
Weekly at Home Stool Collection	✓	✓		✓	✓	✓	✓	
Clinic Stool Collection	✓			✓		✓	✓	
Blood Draw *	✓	✓		✓	✓	✓	✓	
Questionnaires								
SF-36	✓			✓		✓	✓	
IBDQ	✓			✓		✓	✓	
MAYO & UCEIS Endoscopic Score		✓				✓		
Mayo Symptom Questionaire	✓			✓		✓	✓	
Plant Species Log	✓			✓	✓	✓		
Urine Pregnancy Test (if applicable)**	✓			✓	✓	✓	✓	
Endoscopic Procedure with Biopsy								
Colonoscopy		✓						
Flexible Sigmoidoscopy						✓		
FMT Treatment***								
Induction (colonoscopy)		✓						
Pick up/Take home frozen capsules		✓		✓	✓			
Review of Medical History	✓	✓		✓	✓	✓	✓	✓
Review of Current Medications	✓	✓		✓	✓	✓	✓	✓
Assessment of Adverse Events	✓	✓	✓	✓	✓	✓	✓	✓

* 13 teaspoons of blood will be drawn at each visit

** Urine pregnancy tests will be completed if the subject is female and is of child bearing age

*** FMT via colonoscopy at time 0 followed by oral fecal capsule intake daily for 12 weeks

**** Timing of scheduled visits can be flexible within 72hrs

6.2 Informed Consent

Please see Appendix III for a complete listing of all consent forms administered to every patient prior to enrollment in the study.

6.3 Investigator and Facilities Data

Form 1572 and Primary Investigator CV included in Appendix IV

7. CHEMISTRY, MANUFACTURING, AND CONTROL INFORMATION

7.1 Drug Substance

Fecal Microbiota Preparation

Contents: Human Stool (active ingredient); Normal non-bacteriostatic saline (0.9%; excipient); USP Glycerol (.99% glycerol in 1% water, excipient); Cocoa Butter (excipient); Gelatin Capsules (excipient)

Drug Master File for this product has been included as part of the supplemental material

7.1.1. The Name and Address of the Drug Manufacturer

OpenBiome

196 Boston Ave.
Suite 1000
Medford, MA 02155
info@openbiome.org
617-575-2201

7.2 Drug Product

Fecal Microbiota Preparation

The fecal microbiota preparation is synthesized from human feces collected from healthy individuals. The sample is deposited into a container placed over a commode and then properly sealed into one LDPE plastic bag (Ri-Pac 2GN or similar) in order to avoid contamination. Collected samples will be transferred to a UV-sterilized biosafety cabinet dedicated to sample processing and no other tasks. The sample will be cleaned with a sporicidal agent and transferred to a sterile filter bag. An autoclaved dilutent consisting of 12.5% glycerol and saline buffer will be added to the filter bag and the sample homogenized in a blender for 60 seconds. They will then be aliquoted into sterile bottles sing disposable, sterile pipettes and the bottles will be frozen at approximately -20ºC and sealed with tamper evident shrink bands.

The oral preparation will consist of an autoclaved or sterile filtered diluent consisting of 50% glycerol and a normal saline buffer (0.90% w/v NaCl in water) that is added to the filter bag of stool at a ratio of 1g stool:1mL diluent. Samples will be blended and aliquoted into sterile bottles using sterile, disposable serological pipettes. The bottles will be capped and frozen immediately at - 20°C. At the time of encapsulation, samples will be thawed completely at room temperature. 550 μL of the material will be transferred into a Size 0 HMCP colored Gelatin capsule (Capsuline, White#20- 1) and stored in a rack on dry ice for immediate freezing. 210μL of warm cocoa butter will be micropipetted to coat the inside of a Size 00 HMCP capsule (Capsuline, White #20- 1), with 70μL aliquoted into the capsule top and 140μL aliquoted into the capsule bottom. Upon sealing the inner capsule inside the outer capsule with cocoa butter, groups of 25 frozen and filled capsules will be transferred to blister packaging (Medi- Dose, MD145/455) or HDPE bottles (eBottles, 1422) for long-term storage.

7.2.1. The Name and Address of the Drug Product Manufacturer

OpenBiome
196 Boston Ave,
Suite 1000
Medford, MA 02155
info@openbiome.org
617-575-2201

7.3 Placebo

Placebo preparations for colonoscopy will be manufactured by OpenBiome and composed of an autoclaved or sterile filtered diluent consisting of 12.5% glycerol, normal saline buffer, and food coloring (AmeriColor, 204, or similar) without addition of human stool.

Placebo capsules will be manufactured by OpenBiome and composed of an autoclaved solution consisting of 80% cocoa butter, 20% glycerol, and brown food coloring (AmeriColor, 204, or similar) without addition of human stool. Contents will be pipetted and packaged into Size 00 acid-resistant, HPMC colored capsules (Capsugel, White #G60CS000753) for oral use.

7.4 Labeling

The product will be stored in glycerol at -20ºC to preserve microbiological activity. Users will

determine dosage and route of administration; however, clinicians will be advised that once the seal on a bottle has been broken, it must be delivered immediately or properly disposed of as a biohazard.

The following information will be included on the product label:

1. Contact information and instructions to immediately report adverse events
2. A unique sample ID and expiration date
3. Allergen warning: 'Caution: may contain food allergens'
4. Contents: Human feces filtered to 330 microns, Sodium Chloride (0.9%), Glycerol (12.5%), and Water
5. Instructions for storage and disposal

A sample label has been provided in Appendix I.

Additionally, the following documentation will be provided to the clinician with each lot of stool:

- Complete copies of all screening results from the donor whose stool is being provided
- Receiving & storage protocols
- Data tracking log
- Adverse event reporting protocols
- Non- response reporting form
- Detailed descriptions of product quality control
- FDA MedWatch Form 3500

7.5 Environmental Assessment

We request a claim for categorical exclusion for this proposed clinical trial as provided for in 21 CFR.25.31(e) in that the drug shipped under this notice is intended to be used in clinical trials in which the amount of waste expected to enter the environment may reasonably be expected to be non-toxic

8. PHARMACOLOGY AND TOXICOLOGY INFORMATION

8.1 Pharmacology and Drug Distribution

The pharmacokinetics and distribution of fecal microbiota preparation need not be relevant since stool contents are neither absorbed nor transmitted through a healthy, intact intestinal wall. As stated previously, fecal microbiota transplantation and stool preparation grew from empirical clinical practice instead of a conventional drug development program. Accordingly, salient non-clinical data is not particularly informative, particularly in context that the human microbiome has co-evolved with its host since birth. Appendix I has the drug label listed for references purposes.

8.2 Toxicology

To date, there has been no toxicity effect relating directly to fecal microbiota transplantation.

8.3 Statement of Compliance with GLP

This is a clinical research project conducted in a clinical setting, outside of a typical laboratory practice.

9. PREVIOUS HUMAN EXPERIENCE

9.1 Previous experience with treatment of *Clostridium difficile* infection

A robust systematic review and meta-analysis of 11 observational studies with a total of 273 CDI patients did not report any adverse events attributable to the FMT material in variable follow-up from 3 weeks to 8 years.[21] However, 3 studies that used upper gastrointestinal FMT indicated that the nasogastric/nasojejunal tube itself could not be ruled out in contributing to a suspected adverse event: upper gastrointestinal bleed, peritonitis, an possibly enteritis.[21]

A multi-center retrospective review (n=77) reported no definite FMT adverse events in follow-up

between 3-68 months (mean 17 months). The authors did report 4 patients developing diseases of potential interest after FMT (peripheral neuropathy, Sjogren's disease, idiopathic thromobocytopenic purpura, rheumatoid arthritis) and 7 non-FMT related deaths. Interestingly, the study noted post-FMT improvement in a patient with pre-existing allergic sinusitis and one with "arthritis", with the type unreported.[24]

Safety outcomes for FMT treatment of CDI have also been reported in immunocompromised patients in a multi-center retrospective review (n=80).[25] In this special group, 12 patients (15%) had a serious adverse event within 12 weeks of FMT, of which 10 were hospitalized. Specifically, 2 deaths occurred within 12 weeks, one a result of aspiration at the time of colonoscopy for FMT, and the other unrelated to FMT (progressive pneumonia). There were 3 deaths reported 6 months after FMT due to chronic, progressive medical conditions unrelated to FMT. There was another procedure-related complication as a subject sustained a superficial mucosal tear during colonoscopy. Importantly, no patients experienced an infection definitively ascribed to FMT, although 2 subjects sustained unrelated infections (influenza, catheter line infection). Self-limited diarrheal illness occurred in 5 patients but no causal organism was identified.[25]

In a small randomized controlled trial (n = 42) for recurrent CDI assessing FMT via duodenal infusion compared to vancomycin and bowel lavage, or vancomycin alone, adverse event were minor and short-lived.[23] In the FMT arm (n=16), minor gastrointestinal symptoms occurred on the day of infusion including belching (n=3), nausea (n=1), abdominal cramps (n=5), and abdominal pain (n=2); however, these symptoms resolved in all patients within 3 hours. In follow-up, constipation occurred in three patients. There were no adverse events attributed to FMT at 10-week follow-up.[23] In another small, open-label, randomized controlled trial (n=20) for recurrent CDI comparing FMT by nasogastric tube versus colonoscopy, no adverse or unexpected events were documented in the 6-month follow-up period.[22]

To date, there have been two anecdotal cases of transient bacteremia potentially attributable to FMT, which was successfully treated with antibiotics. One case involved a 52-year-old man with Hepatitis C and alcoholic liver cirrhosis admitted for pneumococcal bacteremia, who subsequently developed severe, complicated CDI including ileus that was non-responsive to standard therapy. The patient underwent FMT via nasoduodenal tube and although diarrhea improved, 4 days after FMT was admitted to the intensive care for septic shock. Blood cultures grew 2 strains of pan-susceptible E. coli, and peritoneal fluid also contained E. coli. The patient was successfully treated with conventional sepsis therapy and improved.

Table 1 in Appendix II has a summary table reporting the safety and efficacy outcomes for FMT in CDI

9.2 Previous experience with the treatment of inflammatory bowel disease

The overwhelming success of FMT in the treatment of CDI has led many investigators to look at FMT as an adjunct treatment for IBD. To date, there have been three published systematic reviews, mostly comprised of case reports and cohort studies. In the latest, most comprehensive review of FMT application in IBD, comprising of 119 patients with varying degrees of IBD, fecal microbiota transplantation was overall well tolerated and without major adverse effects. Table 2 in Appendix II has a summary of the safety and clinical efficacy.

Varying degrees of fever, with or without rigors, abdominal tenderness, and CRP elevation after FMT were reported in 11 patients (15%) from four studies. The severity of the fever described varied, was often self-limited and only lasted a few hours following therapy. In only rare cases did the fever require anti-pyretic medications. Almost all other reported events included vague,

nonspecific gastrointestinal symptoms like gas, nausea, bloating and diarrhea. One study reported a patient with pancreatitis of unknown origin during follow-up. No major adverse events were observed in the randomized controlled trial

10. ADDITIONAL INFORMATION

10.1 Drug Dependence and Abuse Potential

There is minimal concern for drug dependence and abuse potential

10.2 Radioactive Drugs

There are no radioactive drugs being used in the study

10.3 Pediatric Studies

There are no pediatric participants in the study. Data supporting fecal microbiota transplantation for recurrent *C. difficile*, inflammatory bowel disease, and other disorders is incredibly limited.

Appendix I: Labels

Fecal Microbiota Preparation

OPENBIOME

Ingredients: Human feces filtered to 330 microns, Sodium Chloride (0.9%), Glycerol (12.5%), Water.
Uses: For use in fecal microbiota transplantation under medical supervision.

Other information: ■ Store immediately at -20°C. ■ Thaw either for 1 hour in a 30°C water bath, 4 hours at room temperature, or overnight at 4°C. ■ Thaw directly before use. ■ After thawing, samples may remain at room temperature for up to 4 hours (or refrigerated for up to 8 hours).
■ Do not refreeze thawed material – thawing and refreezing may compromise viability. ■ Use before expiration date indicated below.

Warnings: ■ May contain food allergens, including nuts.
■ Immediately dispose of unused material as hazardous waste.
■ Screened for common infectious agents (consult quality metrics for details).

FMT is an investigational therapy that has not been approved by the FDA. Immediately report any adverse events to safety@openbiome.org. Produced by OpenBiome at 196 Boston Ave, Suite 1000, Medford, MA.

Expiry Date

Appendix II

Table 1: Efficacy and safety outcomes for FMT in CDI

Author & Year	Study size	CDI Type	FMT route & type*	Efficacy outcomes after up	Safety outcomes***
Youngster et al. (2014)	20	rCDI	CsC or NGT (frozen)	90% (1-2x)<br><br>70% (after 1x only) 100% (3 children)	AE: abdominal discomfort, bloating in 20% of patients.
Van Nood et al. (2013)	16	rCDI	NDT	94% (1-2x)<br><br>81% (after 1x only) Non-FMT arms stopped early for benefit.	AE: constipation (19%), unrelated urinary tract infection (6%) and culture-negative fever during hemodialysis (6%).
Lund-Tonnesen et al. (1998)	18	mix	CsC, G-tube (frozen)	83%(1x)	No ‘‘complications during or after’’ FMT.
Hamilton et al. (2012)	43	rCDI and IBD	CsC (fresh or frozen)	95%(1-2x) Patients who also had IBD: statistically same.	AE: irregular BM, more flatulence in ~33% of patients for ≤2 weeks.
Mattlia et al. (2012)	70	mix	CsC	94% (1x)	No related AEs.
Kelly et al. (2012)	26	rCDI	CsC	92% (1x)	AE: loose or irregular BM in 11.5% of patients; mild & temporary effect.
Mellow et al. 2011[37]	13	mix	CsC	92% (1x)	No comment made. Note: 1 CDI recurrence at 7 months.
Garborg et al. 2010[38]	40	rCDI	CsC, gastroscopy	90% (1-2x)	No comment made about responder AEs.
Rohlke et al. 2010[39]	19	rCDI	CsC	100% (1-2x)	No AEs.
Yoon et al. 2010[40]	12	mix	CsC	100% (1x)	No AEs.

Kassam et al. 2012[41]	27	mix	Enema	93% (1-2x)	No AEs.
Silverman et al. 2010[42]	7	rCDI	Enema	100% (1x)	One patient developed IBS symptoms.
Paterson et al. 1994[43]	7	mix	"rectal tube"	100% (multiple infusions)	No comment made regarding AEs.
Kelly et al. 2014[25]	80	mix and also IC	Mostly lower GI; varied	89% (1-2x, IC), incl. 5 pediatric patients 94% (1-2x, sub-group with concurrent IBD)	SAEs: 1 procedure-related, 1 severe abdominal pain, 4 IBD flares. See discussion. AEs: 4 related, 5 possibly
Polak et al. 201144	15	rCDI	NJT	87% (1-2x)	SAEs: 1 procedure-related.<br><br>No comment on other AEs.
MacConnachie et al. 200945	15	rCDI	NGT	73% (1-2x)	No material-related AEs. One upper GI hemorrhage, may be procedure-related.
Aas et al. 200346	18	rCDI	NGT	83%(1x)	SAEs related to procedure or co-morbidities only: peritonitis, pneumonia.

Notes:

* FMT product is fresh as opposed to frozen unless stated otherwise.
**Note that follow-up time for examining efficacy was variable. Most studies continued for at least 1.5-2 months, and some for years, but at least one continued for only 2-3 weeks.
*** This column focuses on responder AEs. Note that patients who were unresponsive to FMT treatment died of rCDI or other non-FMT-related causes in several studies.

Abbreviations:

CDI form: rCDI = recurrent Clostridium difficile infection; mix = mix of recurrent (repeated), refractory (not responding to treatment), and severe (e.g., hospitalization) CDI.
CDI form and also Efficacy: IBD = inflammatory bowel disease; IC = immunocompromised. FMT route: CsC = colonoscopy; G-tube = gastrotomy tube; NGT = nasogastric tube; NDT = nasoduodenal tube; NJT = nasojejunal tube.
Safety: SAE = serious adverse event; BM = bowel movement; IBS = irritable bowel syndrome

Table 2: Efficacy and safety outcomes for FMT in IBD

Author & Year	Study Size	IBD type	FMT & Route	Efficacy outcomes	Safety outcomes
Vermeire et al. (2012)	4	CD	200g; NJ	No patient reported improvement	3 patients had high fever and abdominal tenderness at day of FMT that disappeared within 2 days
Kunde et al. (2013)	10	UC	90-113g/ 250ml; Enema	33% no improvement<br>33% symptom improvement without remission<br>33% clinical remission	1 patient had fevers/rigors 3h after FMT; resolved with Tylenol and Benadryl<br>1 patient had low grade fever; self-resolved<br>3 patients had fatigue<br>9 patients had non-specific GI symptoms
Kump et al. (2013)	6	UC	100-150g/200-350ml; C-scope	1 patient deteriorated<br>50% no improvement<br>33% symptom improvement without remission	1 patient had self-limiting fever + diarrhea day 1 post FMT; slef limited
Angelberger et al. (2013)	5	UC	60g/250ml; NJ + 6-22g/100ml Enema	2 patients deteriorated<br>2 patients without improvement<br>1 patient with symptomatic improvement without clinical remission	Fever + CRP elevation in 5 patients<br>NJ tube irritation in 5 patients<br>Flatulence in 2 patients<br>Vomiting in 1 patient
Suskind et al. (2014)	9	CD	NR; NG	90% clinical remission<br>10% symptom improvement without remission	Bloating in 3 patients dat after FMT; self-limited
Landy et al. (2013)	5	UC	30g/250ml; NG	No results reported	NR
Zhang et al. (2013)	16	CD	NR, EGD	75% clinical remission<br>3 patients with symptomatic improvement without remission<br>1 patient with no improvement	Diarrhea in 5 patients 3 hours after FMT; self-limited
Damman et al. (2014)	8	UC	NR; C-scope	30% clinical remission<br>1 patient deteriorated<br>57% no improvement	NR
Vaughn et al. (2014)	9	CD	50g/250ml; C-scope	45% clinical remission<br>33% without improvement<br>22% with symptomatic improvement without remission	No complications in first 4 weeks following FMT

Appendix III:
Informed Consent V6, 5/15/17
Pilot/Control Consent V4, 1/24/17

Informed Consent

for

Fecal Microbiota Transplantation for Ulcerative Colitis

Principal Investigator: Peter L. Moses, MD

You are being invited to take part in this research study because you have been diagnosed with Ulcerative Colitis (UC) and have reported to your doctor that you have mild to moderate symptoms of UC. This is a voluntary study that is being conducted to investigate the use of Fecal Microbiota Transplantation (FMT) as an aid to the treatment for active Ulcerative Colitis. This is a randomized control trial and there is a 50% chance that you will receive placebo (sham) FMT. This study is being conducted by the University of Vermont at the UVM Medical Center.

We encourage you to ask questions and take the opportunity to discuss the study with anybody you think can help you make this decision.

<u>Why is This Research Study Being Conducted?</u>

The purpose of this study is to evaluate the effectiveness of FMT for treating patients with mild to moderate UC. Even with the expanding choices of medication for UC, physicians and patients are still in search of highly effective and safe medications with minimal side effects. The FDA currently recognizes FMT as an option in the treatment of a bacterial infection called *Clostridium difficile,* if the infection becomes chronic. In this setting, FMT has been proven to be effective and safe. FMT has not been approved by the FDA for the treatment of UC.

The investigators conducting this study have proposed it based on data suggesting a role for the alteration of gut microbes in treating UC. Alteration of gut microbes can be accomplished by “transplanting” fecal material, which contains a highly complex and dense community of healthy microbes, including bacteria, fungi and viruses. This collection of microbes is referred to as a “microbiome”. The change in this microbiome brought forth by fecal transplantation has been established as the explanation in successfully treating *Clostridium difficile* diarrhea. The purpose of this study is to examine whether similar alterations of the microbiome can help treat UC.

In order to study the effectiveness of FMT in UC, this study is designed as a placebo-controlled study. This means that one group is randomized to receive a real fecal transplant and another group is randomized to receive placebo treatment. Placebo treatment does not contain actual fecal material. There is a 50% chance that you will be selected for either the placebo group or

the FMT group. This selection is randomized by a computer. All subjects will be continued on their prescribed UC medications throughout the study.

Some research suggests that diet may have an important impact on the bacteria of your gut. In order to study this connection we are asking our study participants to write down the number of different plant species they eat each day.

How Many People Will Take Part In The Study?

There will be approximately 40 people participating in this study at The University of Vermont Medical Center.

What Is Involved In The Study?

Screening Visit:

At the first visit, the doctor or his associate in charge of the study will explain the study to you and you will be given this document, the informed consent form. If you decide to participate in this study and sign this form you will be asked to fill out three questionnaires called IBDQ, SF-36 and a MAYO Score. These questionnaires will ask about your symptoms associated with your UC. You will also be asked several questions with respect to your medical history, current medications and allergies. A plant species log will also be provided to you to document the number of different plant species you consume over the next 4 weeks.

If you choose to participate, you will also be asked to provide a stool and blood sample during this visit. These samples will used to test the level of inflammation in your gut and systemically.

At this visit and in preparation for the FMT (procedure visit) you will be asked to start taking seven days of two antibiotics orally. The two antibiotics are ciprofloxacin 250mg to be taken twice a day and metronidazole 500mg to be taken three times a day. The purpose of taking antibiotics is to “clear your gut” which some evidence suggests helps the landing of new bacteria, thereby maximizing the FMT treatment’s effect. These antibiotics will not be billed to your insurance; these medications will be provided to you and paid for by the research funding.

There are no dietary restrictions related to food or other medications in this study.

You should remain taking your current UC medications as previously prescribed throughout the entire study. If you are prescribed any new medications during this study, you will be asked to disclose that information to the investigators.

You will be sent home with stool collection kits. These kits are labeled with a study number, but have no personal identifying information. Each kit includes one collection vial, directions, and a prepaid package for shipping via standard US mail. We ask that you collect 3 stool samples at home before you begin taking antibiotics and then 1 stool sample after you finish your course of

antibiotics, but before you come in for your colonoscopy. You will send all of the samples to the research team by mail.

Finally, you will be asked to provide a urine sample for a urine pregnancy test if you are a female of child-bearing age. You will be counseled to avoid pregnancy for the duration of the study. In the event of an unplanned pregnancy, you will be asked to cease all study medications and will be followed by our medical team along with your primary care physician/obstetrician for the duration of the pregnancy.

This screening visit should take one hour.

Procedure Visit:

Starting the night before the procedure you will be asked to drink a colonic preparation called polyethylene glycol, also known as GoLYTELY (this is the same colonic cleanse used routinely in preparation for colonoscopy). This is to be taken orally in two sessions—2 liters the night prior to FMT delivery and 2 liters the morning of FMT delivery. A rate of 1 glass (8 oz.) every 10 minutes is recommended until completed. The procedure will take place 7 days after the screening visit. During this visit you will also be asked to provide a blood sample.

After this is obtained you will then be sedated for the procedure with a medication called Versed, and a pain medication called Demerol or a medication called fentanyl. If you have any allergies to these medications or medications like these drugs, you will be given an alternative medication to sedate you during this procedure. These medications will be given in small amounts and an exact dosage is different for each individual. This information will be reviewed prior to the procedure and consent will also be obtained prior to the procedure

In the first part of the colonoscopy, your doctor will examine the surface of your colon by using the small camera attached to the end of the colonoscope (a flexible tube inserted into your bowel). Biopsies will be taken. During the second part of the colonoscopy, your doctor will perform the first dose of the fecal transplant by delivering the instilled fecal material or a placebo solution in the terminal ileum (the last part of the small intestine) and in the right colon. You will be instructed to lie on your left side after the procedure for one hour.

Please allow approximately four hours for the procedure visit. As with any colonoscopy, due to the sedation administered, a ride home will need to be provided from someone other than yourself.

The remainder of your FMT treatment will be the same material you received during the colonoscopy but in a capsule that you will take orally. If you are randomized to the placebo group, you will receive placebo capsules. After the colonoscopy visit is complete you will take home 4 weeks' worth of frozen fecal or placebo capsules in a cooler with dry ice. Information about the proper handling and disposing of dry ice will be provided to you. You will be asked to complete a tracking sheet each time you take your capsule.

In order to keep the FMT capsules stable they must remain frozen at all times and therefore **any planned travel will not be allowed during the capsule maintenance period**. We understand that there may be unplanned family or personal emergencies. In the event of an emergency, please contact the study team for further instructions.

You will be given a 4-week supply of at-home stool collection kits. We ask that the first sample you collect be your 2nd or 3rd bowel movement after the procedure and then each additional sample be collected weekly thereafter.

A follow-up phone call will be placed to you 24 hours after the FMT or placebo colonoscopy to assess any side effects or adverse events.

Oral FMT or PLACEBO, Weeks 1-4:

You will take 4 weeks of oral FMT or placebo capsules daily. You will be asked to take 1 capsule per day anytime you wish, as long as the timing is consistent throughout the whole study. The FMT or placebo capsules must be kept inside a Styrofoam cooler with icepacks and the dry ice it came with in your home freezer. It is important to keep the capsules inside the cooler because the temperature in your freezer changes over time and this will help keep the capsules frozen. You will be asked to record your intake of these capsules by filling out a tracking log. If you miss doses, this should be recorded honestly.

After you collect a sample from your second or third bowel movement following the procedure, we ask that you collect a weekly stool sample at home. You can collect the samples whenever you like, however we ask that you try to collect it on the same day each week. You will send the sample to our research team through the mail in a provided, prepaid, envelope.

4 Week Visit:

At the end of 4 weeks you will be asked to come back to the UVM Medical Center to drop off the empty cooler with your tracking sheet and pick up your second 4 week batch of FMT capsules. You will also be asked to give a blood sample, stool sample and to fill out three questionnaires to monitor your symptoms and quality of life. This will help us assess for any changes since starting your FMT treatment. You will also be asked several questions with respect to your medical history, current medications and updated allergies. Any adverse events will be recorded. If applicable, you will have to submit another urine specimen for a urine pregnancy test. Afterwards, you will take home a new cooler with ice packs and dry ice containing the second 4 weeks' worth of FMT or placebo capsules. You will also be given a 4-week supply of at-home stool collection kits. Another plant species log will also be provided to you to document your plant species over the next 4 weeks.

Please allow up to one hour for this visit.

Oral FMT or PLACEBO, Weeks 4-8:

You will take a second 4 weeks of oral FMT or placebo capsules daily. You will be asked to take 1 capsule per day anytime you wish, as long as the timing is consistent. You will again be asked to record your intake of these capsules by filling out a tracking log. If you miss doses, this should be recorded honestly. Again, these capsules must be kept in a cooler with icepack and dry ice inside your home freezer.

You will be asked to continue to collect a weekly stool sample at home during this time period. You can collect the samples whenever you like, however we ask that you try to collect it on the same day each week. You will send the sample to our research team through the mail in a provided, prepaid, envelope.

8 Week Visit:

At the end of 8 weeks you will be asked to come back to the UVM Medical Center to drop off the empty cooler with your tracking sheet and pick up your third 4 week batch of FMT capsules. You will also be asked to provide a blood sample and, if applicable, a urine sample for a urine pregnancy test. Afterwards, you will take home a new cooler with ice packs and dry ice containing the third 4 weeks' worth of FMT or placebo capsules. You will also be given a 4-week supply of at-home stool collection kits. Another plant species log will also be provided to you to document your plant species over the next 4 weeks.

Please allow up to one hour for this visit.

Oral FMT or PLACEBO, Weeks 9-12:

You will take your third 4 weeks of oral FMT or placebo capsules daily. You will be asked to take 1 capsule per day anytime you wish, as long as the timing is consistent. You will again be asked to record your intake of these capsules by filling out a tracking log. If you miss doses, this should be recorded honestly. Again, these capsules must be kept in a cooler with icepack inside your home freezer.

You will be asked to collect a weekly stool sample at home during this time period. You can collect the samples whenever you like, however we ask that you try to collect it on the same day each week. You will send the sample to our research team through the mail in a provided, prepaid, envelope.

12 Week Visit:

At the end of 12 weeks you will be asked to come back to the UVM Medical Center to drop off the empty cooler with your tracking sheet. You will again be asked to give a blood and stool sample, and fill out three questionnaires to monitor your symptoms and quality of life to assess for any changes since completing your FMT treatment. You will also be asked several questions with respect to your medical history, current medications and updated allergies. Any adverse events will be recorded. If applicable, you will have to submit another urine specimen for a urine pregnancy test. Another plant species log will also be provided to you to document your plant species over the next 4 weeks.

At this visit you be asked to participate in a procedure called a flexible sigmoidoscopy. This procedure is used to see inside the sigmoid colon and rectum. The sigmoid colon is the area of the large intestine closest to the rectum. There is no GoLytely prep for a sigmoidoscopy. The only preparation will be a Fleet enema (mineral oil) provided at your visit. The enema will be gently inserted into your rectum and injected into your lower colon by a healthcare professional in the clinic exam room. This enema allows for adequate visualization. Once the enema is completed the doctor will place a flexible tube with a camera on its end to view the rectum and colon. The physician will take small biopsies and examine them under a microscope to look for evidence of inflammation. This flexible sigmoidoscopy procedure is done in the clinic as an outpatient. No sedation is used for this procedure. Therefore, you do not need to have a ride provided by someone else. Please allow up to one hour for this visit. This procedure will not be billed to your insurance and will be paid for by the research team.

18 Week Visit:

At the end of 18 weeks you will be asked to come back to the UVM gastroenterology clinic to once again provide a blood and stool sample, and fill out three questionnaires to monitor your symptoms and quality of life to assess for any changes since completing your FMT or placebo treatment 6 weeks prior. You will also be asked several questions with respect to your medical history, current medications and updated allergies. Any adverse events will be recorded. You will be given 3 last stool sample collection kits for use at home. If applicable, you will have to submit another urine specimen for a urine pregnancy test. Please allow up to one hour for this visit.

Stool Collection:

Should you choose to take part in this study, you will be asked to supply your own stool samples. These samples will provide information about your own gut bacteria, known as your unique “microbiome”. You will be provided with a kit to collect your stool at home. The kit will be shipped via US Postal Service to a laboratory named OpenBiome in Boston, MA for evaluation of your unique microbiome. The stool collection will occur at baseline (3 samples after your screening visit before you begin antibiotic therapy and 1 sample after finishing antibiotic therapy but before colonoscopy), weekly throughout the duration of the study (12 weeks) and again (3

samples) at the 18-week follow-up time point. Instructions will be provided with this kit and we will review the process with you at each of your visits. Your samples will only be identifiable by a unique ID number. No personal information will be detectable on the sample or mailer.

36 Week Phone Call:

At the end of 36 weeks you will receive a phone call from a member of the study team to review your medical history, current medications, and any adverse events. This phone call is approximately 6 months after your last FMT or Placebo capsule ingestion.

Bio Banking and Implications

Portions of the stool samples you provide as part of this study that are not required for routine diagnosis or treatment may be used now or in the future for research purposes. These samples may be used to learn more about how cancer or other diseases develop and/or may result in new products, tests or discoveries. In some instances, these may have potential commercial value. These samples to be kept for research purposes will be obtained only at the same time as your regular procedures are performed; you will not have to undergo any special procedures for this purpose. There will be no additional charge, and you will not receive any payment or financial benefit from any products, tests or discoveries. You may also be asked in the future if you are willing to be in additional research studies. You will not be told the results of any future research. Participation in this extra research is voluntary, and if you choose not to allow the extra research it will in no way affect your care on the main study. You may at any time contact the researchers to request that your samples be withdrawn from research use, and any identifiable samples still in their possession will be destroyed. Please indicate whether you are willing to allow this extra research by initialing one of the lines at the end of the form.

	Screening Visit *(Day -7)*	Procedure Visit *(Day 0)*	Follow-Up Phone Call (24hrs after FMT)	4 Week Visit *(Day 28+-72hrs.)*	8 Week Visit *(Day 56+-72hrs.)*	12 Week Visit *(Day 84 +-72hrs.)*	18 Week Visit *(Day 126 +-72hrs.)*	36 Week Phone Call *(Day 264 +-72hrs.)*
Weekly at Home Stool Collection	✓	✓		✓	✓	✓	✓	
Clinic Stool Collection	✓			✓		✓	✓	
Blood Draw *	✓	✓		✓	✓	✓	✓	
Questionnaires								
SF-36	✓			✓		✓	✓	
IBDQ	✓			✓		✓	✓	
MAYO & UCEIS Endoscopic Score		✓				✓		
Mayo Symptom Questionaire	✓			✓		✓	✓	
Plant Species Log	✓			✓	✓	✓		
Urine Pregnancy Test (if applicable)**	✓			✓	✓	✓	✓	
Endoscopic Procedure with Biopsy								
Colonoscopy		✓						
Flexible Sigmoidoscopy						✓		
FMT Treatment***								
Induction (colonoscopy)		✓						
Pick up/Take home frozen capsules		✓		✓	✓			
Review of Medical History	✓	✓		✓	✓	✓	✓	✓
Review of Current Medications	✓	✓		✓	✓	✓	✓	✓
Assessment of Adverse Events	✓	✓	✓	✓	✓	✓	✓	✓

* 13 teaspoons of blood will be drawn at each visit

** Urine pregnancy tests will be completed if the subject is female and is of child bearing age

*** FMT via colonoscopy at time 0 followed by oral fecal capsule intake daily for 12 weeks

**** Timing of scheduled visits can be flexible within 72hrs

Where does the FMT stool come from?

Donors who give their stool for FMT undergo extensive screening by the non-profit organization, OpenBiome. Donors do not have a history of Irritable Bowel Syndrome (IBS), constipation, or inflammatory bowel disease (IBD). Donors are screened for diseases like HIV, Hepatitis B and C, syphilis, gonorrhea, chlamydia, stool parasites, and bacteria like *Clostridium difficile*, *Shigella, Salmonella, Campylobacter, and Escherichia coli.* OpenBiome currently supplies stool for the treatment of a bacterial infection called *Clostridium difficile* at The University of Vermont Medical Center.

What Are The Risks and Discomforts Of The Study?

Colonoscopy is a safe procedure associated with a complication rate in the range of approximately 1 in 1,000. Since conscious sedation is administered, most people will experience very little discomfort, approximating a digital rectal exam. The procedure can be stopped at any time if the individual's comfort is compromised. The associated "bowel prep" with Go-LYTELY for a colonoscopy may include discomfort, including bloating, abdominal pain, nausea and vomiting. The "prep" for a sigmoidoscopy is an enema, this is where a nurse will gently insert the enema tip into your rectum and inject the liquid into your lower colon. This may cause some very mild discomfort but is a safe procedure without risk when performed by a trained health care professional.

Inherent risks of any colonoscopy include infection, perforation of the bowel wall, or bleeding. These risks can occur at a rate of 1 per 1,000 colonoscopies. Other extremely rare, but serious or possibly fatal risks include: difficulty breathing, heart attack, and stroke. Also, sedation and pain relieving medications may be given to minimize discomfort and relax you for the procedure. These medications may cause localized irritation and/or a drug reaction. By signing this informed consent form you agree to these risks. You also will be unable to drive the remainder of the day and should not have plans after the procedure.

The flexible sigmoidoscopy has minimal risk of slight bleeding from the biopsy site, and possible infection and/or perforation. This complication rate is less than 1 in 10,000.

The risks of the colonoscopy and sigmoidoscopy will be identical in both the FMT group and the placebo group.

FMT has not yet been studied in pregnant women. While the potential risks are not known, we require the use of contraception for the entire duration of the study period. If you wish to become pregnant during the study period, please discuss this with the study team.

Mild fever and abdominal pain following FMT have been described. FMT has only been studied in a small number of patients with active UC. Therefore, the actual risks of FMT in this setting are not yet known. The risks of 12 weeks of daily oral FMT capsules are also not known.

If you choose to participate in this research study, the risks associated with the procedures will be the same in both the FMT and placebo groups. The risks of FMT, however, can only be applied to the group receiving FMT (not placebo). Importantly, if you are assigned to the placebo group, you will be subjected to the risks associated with pretreatment with antibiotics, as well as those associated with sigmoidoscopy, without the possible benefit of this treatment. Regardless of the group to which you are assigned, you will be monitored for an hour after the procedure for any adverse events and will receive a telephone call the day after to assess for any complications. If you choose to not participate in this research study, you will not be exposed to any of the risks associated with the study other than the risks involved with the initial colonoscopy.

<u>What Are The Benefits of Participating In The Study?</u>

Current UC treatment options are limited. Increasing dosages and new classes of medications come with inherent risk and variable success. Patients have mild to moderate symptoms associated with UC despite standard treatment should weigh the risks and benefits of additional treatments, including experimental therapy. If proven effective, a role for FMT in the standard treatment of UC for some patients may be identified. As FMT for UC is still investigational, it is possible that there may be no benefit to you.

<u>What Other Options Are There?</u>

You do not need to participate in this study to continue to receive medical care at UVM Medical Center. You may instead choose to continue treatment for your UC with alternative medications.

<u>Are There Any Costs?</u>

Colonoscopy is used for prognostic and surveillance purposes of UC. This procedure is considered to be part of the standard of care when evaluating symptoms, as the findings can be helpful in confirming the diagnosis and evaluating the severity and extent of your UC. Therefore, the cost of the procedure should be covered by insurance.

The FMT solution/treatment given during the colonoscopy and the daily 12 week capsules will be provided by the study team at no cost to you. The colonoscopy to deliver the FMT solution/treatment will be billed to your insurance.

The flexible sigmoidoscopy at week 12 will be paid for by the investigator’s research funds. There will be no cost to you for this procedure.

Antibiotic treatment (Ciprofloxacin and Metronidazole) will be provided free of cost. You will remain on your currently prescribed UC medication regimen throughout the course of the study, and the research team will not pay for these medications.

What Is the Compensation?

There is no financial compensation provided to you for your participation in this study.

Can You Withdraw or Be Withdrawn From This Study?

You may choose to discontinue treatment at any time. If you choose to decline your participation in this study it will not affect your care at UVM Medical Center in any way. Should your disease become worse, should side effects become very severe, should new scientific developments occur that indicate the treatment is not in your best interest, or should your physician feel that this treatment is no longer in your best interest, the treatment will be stopped. In addition, the researcher may discontinue your participation in this study at any time.

What About Confidentiality of Your Health Information?

What health information will be used and disclosed for this study?

The health information we plan to collect for this study is listed below.

- Medical history and examinations.
- Information that identifies you, such as your name, address, age, and sex.
- Reports from hospital and clinic visits
- Laboratory and other test results
- X-ray and other images and reports
- Lists of medications you are taking
- Responses to health surveys and questionnaires
- Reports of testing for infectious diseases, including HIV

Who is disclosing your health information for this research study?

- The University of Vermont Medical Center
- Other doctors' offices and hospitals where you may receive medical care while this study is active.

Who will use your health information in this study?

Our research team will use your health information. We may also share it with those who assist with the conduct of the research or oversight of the activities for this study. The representatives from the institutions, organizations, and agencies are listed below.

- The University of Vermont and its Committees on Human Research
- The University of Vermont Medical Center
- Officials from agencies and organizations that provide accreditation and oversight of research
- The company, OpenBiome, who provides the drugs for this research project
- Other researchers that help with this study at Massachusetts Institute of Technology
- Federal and state agencies that oversee or review research information, such as the U.S. Food and Drug Administration (FDA), the Department of Health and Human Services, the National Institutes of Health, and public health and safety authorities
- Your health insurer, for portions of the research and related care that are considered billable

Your health information is protected by a federal law called the Health Information Portability and Accountability Act (HIPAA). Once your health information is shared outside of the University of Vermont Medical Center, we cannot guarantee that these laws will continue to apply. As a result, your health information could be further disclosed for other purposes. In the absence of a Certificate of Confidentiality, it is also possible for a court or other government official to order the release of study data. The confidentiality of your health information cannot be guaranteed if you agree it may be used in this study.

How long will your health information be used for research?

Your permission to use your health information will not end until the study is completed. During this study, you will not have access to study data. You may ask for your data once study activities are complete. You have a right to receive a copy of the information in your medical record at any time.

What if you decide not to give permission for research use of your health information?

If you decide not to allow the use and disclosure of your health information, you may not take part in this study. Your decision will have no effect on your current or future medical care.

If you later choose to stop taking part in this study in the future, you may cancel permission for the use of your health information. You should let the research team know that you are cancelling your permission. A member of the research team will assist you in making your

decision effective. The study will continue to use the health information already collected for the study before you cancelled your permission.

Who can answer your questions about the use and disclosure of your health information?

If you have questions or concerns about the use and disclosure of your health information, you should ask a member of the study team at (802) 847-2554 or the Privacy Officer at The University of Vermont Medical Center, Inc., at (802) 847-2667.

Safeguarding Your Health Information

A record of your progress will be kept in a confidential form at the Office of Clinical Trials Research. The security of your record will be maintained by the research team. The results of this study may eventually be published and information may be exchanged between medical investigators, but patient confidentiality will be maintained.

If your record is used or disseminated for government purposes, it will be done under conditions that will protect your privacy to the fullest extent possible consistent with laws relating to public disclosure of information and the law-enforcement responsibilities of the agency.

Clinical Trials Registration

A description of this clinical trial will be available on *http://www.ClinicalTrials.gov*, as required by U.S. Law. This Web site will not include information that can identify you. At most, the Web site will include a summary of the results. You can search this Web site at any time.

What Happens If You Are Injured?

UVM Medical Center Policy

If you are injured or become ill as a result of being in this research, The UVM Medical Center, the hospital partner of the University of Vermont, will provide reasonable and usual medical care for that injury or illness. There will be no cost to you if the conditions listed below apply to your injury or illness. These conditions are:

1. The investigator, in consultation with the study sponsor, determines that your injury or illness results from the research and not from your underlying condition or its usual treatment.
2. You let the investigator know about the injury or illness when you first notice it; and
3. You follow medical advice about proper treatment options for the injury or illness.

The UVM Medical Center may claim payments for your medical treatment directly from the study sponsor or your insurance company when these payments are allowed.

For an injury or illness that results from being in this study, the University of Vermont and The UVM Medical Center will not offer you any other payments, such as lost wages or expenses, except for your medical care. Even though you may receive medical care at no cost to you under certain conditions if you are in this study, the UVM Medical Center and the University of Vermont do not admit to any responsibility for an injury or illness that results from being in the study.

If you agree to take part in this study and you sign this consent form, you are not giving up any of your legal rights.

You may contact Dr. Peter Moses, the investigator in charge of this study, at (802) 847-2554 for more information about this study. If you have any questions about your rights as a participant in a research project or for more information on how to proceed should you believe that you have been injured as a result of your participation in this study you should contact the Director of the Research Protections Office, at the University of Vermont at 802-656-5040.

Statement of Consent
You have been given and have read or have had read to you a summary of this research study. Should you have any further questions about the research, you may contact the person conducting the study at the address and telephone number given below. Your participation is voluntary and you may refuse to participate or withdraw at any time without penalty or prejudice to your present and/or future care.

You agree to participate in this study and you understand that you will receive a signed copy of this form.

______ *I do not want my tissue, stool, and blood samples used for any research or tests other than those needed for the main research study.*

______ *The researchers may keep my extra tissue, stool, and blood samples for future research.*

______ *I am willing to be contacted in the future about any additional research studies.*

____
Signature of Subject Date

This form is valid only if the Committees on Human Research's current stamp of approval is shown below.

____
Name of Subject Printed

____
Signature of Principal Investigator or Designee Date

____
Name of Principal Investigator or Designee Printed

Peter L. Moses, MD
Gastroenterology & Hepatology
The University of Vermont Medical Center
111 Colchester Ave.
Burlington, VT 05401
(802) 847- 2554

Informed Consent
for
<u>Control Specimen Collection</u>

Fecal Microbiota Transplantation for Ulcerative Colitis

Principal Investigator: Peter L. Moses, MD

PURPOSE

We invite you to participate in a research study being conducted at The University of Vermont Medical Center. The purpose of this study is to investigate potential changes in mucosal immune response in humans with ulcerative colitis. You are being asked for your participation because you are undergoing an endoscopic procedure for one of the following conditions: you are a healthy individual undergoing colon cancer screening or you are a patient with Inflammatory Bowel Disease (IBD) and are undergoing a diagnostic or cancer-screening colonoscopy.

PROCEDURE

You are being asked to allow the physician performing your colonoscopy to remove a small amount of gastrointestinal tissue by pinch biopsy for a clinical trial. This is a technique that is frequently used in colonoscopy. The tissue removed will be used for research at The University of Vermont Medical Center.

Using standard endoscopic biopsy forceps, we will remove a piece of the gastrointestinal lining that is approximately the size of a rice grain. We will obtain three biopsies in order to perform our tissue analysis. Obtaining biopsies in this limited manner will add less than three minutes to the time required for your scheduled procedure. Tissue samples will be processed in the laboratory using a number of methods to assess levels of different immune cells present at the mucosal surface. Your samples will be used as controls in this study.

If you are a healthy (non-IBD) patient undergoing screening colonoscopy you may also be asked to provide a peripheral blood sample (blood draw) to be used for research at the University of Vermont Medical Center. Approximately 20mL will be collected.

RISKS AND DISCOMFORTS

A colonoscopy is a safe procedure associated with a complication rate of approximately 1 in 1,000. Serious complications are extremely unlikely and will be described in further detail by the physician prior to your procedure regardless

of your participation in this study. The biopsy process is painless, and the risk of infection does not increase with biopsies. Although it is possible that pinch biopsy sites may bleed, significant bleeding from these sites is highly unlikely. Bleeding is encountered in less than 5 percent of individuals undergoing this procedure, and in all of these cases bleeding stopped on its own without treatment. There are no significant major complications associated with the biopsy acquisition that would add to the risks of your procedure. Therefore, the acquisition of tissue for this study will not increase the risk, cost or discomfort associated with this procedure.

A peripheral blood draw is a safe procedure commonly used to obtain blood for routine medical testing. Some individuals may experience pain at the blood draw site, bruising and/or soreness.

BENEFITS

There is no direct benefit for participating in this study.

FINANCIAL COMPENSATION

You will not receive financial compensation as a result of your participation in this study.

COSTS

There are no additional costs to you or your insurance company associated with participation in this study. The financial responsibility to you or your insurance company for the procedure will not change as a result of the study.

ALTERNATIVES

Your alternative is to choose not to participate in this study and proceed with your colonoscopy as planned.

CAN YOU WITHDRAW FROM THE STUDY?

Participation in the study is purely voluntary and you can refuse participation without penalty or prejudice to your present or future medical care.

WHAT ABOUT CONFIDENTIALITY OF YOUR HEALTH INFORMATION?

What health information will be used and disclosed for this study?

The health information we plan to collect for this study is listed below.

- Medical history and examinations
- Information that identifies you, such as your name, age.
- Laboratory and other test results
- Tissue samples

Who is disclosing your health information for this research study?

- The University of Vermont Medical Center

- Other doctors’ offices and hospitals where you may receive medical care while this study is active.

Who will use your health information in this study?
Our research team will use your health information. We may also share it with those who assist with the conduct of the research or oversight of the activities for this study. The representatives from the institutions, organizations, and agencies are listed below.

- The University of Vermont and its Committees on Human Research
- The University of Vermont Medical Center
- Officials from agencies and organizations that provide accreditation and oversight of research
- The company, OpenBiome, who provides the drugs for this research project
- Other researchers that help with this study at Massachusetts Institute of Technology
- Federal and state agencies that oversee or review research information, such as the U.S. Food and Drug Administration (FDA), the Department of Health and Human Services, the National Institutes of Health, and public health and safety authorities
- Your health insurer, for portions of the research and related care that are considered billable

Your health information is protected by a federal law called the Health Information Portability and Accountability Act (HIPAA). Once your health information is shared outside of the University of Vermont Medical Center, we cannot guarantee that these laws will continue to apply. As a result, your health information could be further disclosed for other purposes. In the absence of a Certificate of Confidentiality, it is also possible for a court or other government official to order the release of study data. The confidentiality of your health information cannot be guaranteed if you agree it may be used in this study.

How long will your health information be used for research?
Your permission to use your health information will not end until the study is completed. During this study, you will not have access to study data. You may ask for your data once study activities are complete. You have a right to receive a copy of the information in your medical record at any time.

What if you decide not to give permission for research use of your health information?
If you decide not to allow the use and disclosure of your health information, you may not take part in this study. Your decision will have no effect on your current or future medical care.

If you later choose to stop taking part in this study in the future, you may cancel permission for the use of your health information. You should let the research team

know that you are cancelling your permission. A member of the research team will assist you in making your decision effective. The study will continue to use the health information already collected for the study before you cancelled your permission.

Who can answer your questions about the use and disclosure of your health information?

If you have questions or concerns about the use and disclosure of your health information, you should ask a member of the study team at 802-847-8655 or the Privacy Officer at The University of Vermont Medical Center, Inc, at (802) 847-2667.

Safeguarding Your Health Information

A record of your progress will be kept in a confidential form at the Office of Clinical Trials Research. The security of your record will be maintained by the research team. The results of this study may eventually be published and information may be exchanged between medical investigators, but patient confidentiality will be maintained.

If your record is used or disseminated for government purposes, it will be done under conditions that will protect your privacy to the fullest extent possible consistent with laws relating to public disclosure of information and the law-enforcement responsibilities of the agency.

Clinical Trials Registration

A description of this clinical trial will be available on *http://www.ClinicalTrials.gov*, as required by U.S. Law. This Web site will not include information that can identify you. At most, the Web site will include a summary of the results. You can search this Web site at any time.

WHAT HAPPENS IF YOU ARE INJURED?

UVM Medical Center Policy

If you are injured or become ill as a result of being in this research, The UVM Medical Center, the hospital partner of the University of Vermont, will provide reasonable and usual medical care for that injury or illness. There will be no cost to you if the conditions listed below apply to your injury or illness. These conditions are:

1. The investigator, in consultation with the study sponsor, determines that your injury or illness results from the research and not from your underlying condition or its usual treatment.
2. You let the investigator know about the injury or illness when you first notice it; and
3. You follow medical advice about proper treatment options for the injury or illness.

The UVM Medical Center may claim payments for your medical treatment directly from the study sponsor or your insurance company when these payments are allowed.

For an injury or illness that results from being in this study, the University of Vermont and The UVM Medical Center will not offer you any other payments, such as lost wages or expenses, except for your medical care. Even though you may receive medical care at no cost to you under certain conditions if you are in this study, the UVM Medical Center and the University of Vermont do not admit to any responsibility for an injury or illness that results from being in the study.

If you agree to take part in this study and you sign this consent form, you are not giving up any of your legal rights.

Contact Information

You may contact Dr. Peter Moses, the Investigator in charge of this study, at 802-847-8865 for more information about this study. If you have any questions about your rights as a participant in a research project or for more information on how to proceed should you believe that you have been injured as a result of your participation in this study you should contact the Director of the Research Protections Office at the University of Vermont at 802-656-5040.

Statement of Consent

You have been given and have read or have had read to you a summary of this research study. Should you have any further questions about the research, you may contact the person conducting the study at the address and telephone number given below. Your participation is voluntary and you may refuse to participate or withdraw at any time without penalty or prejudice to your present and/or future care.

You agree to participate in this study and you understand that you will receive a signed copy of this form.

____ ___________________________
Signature of Subject Date

This form is valid only if the Committees on Human Research's current stamp of approval is shown below.

____
Name of Subject Printed

____
Signature of Principal Investigator or Designee Date

____
Name of Principal Investigator or Designee Printed

Peter L. Moses, MD
Gastroenterology & Hepatology
The University of Vermont Medical Center
111 Colchester Ave.
Burlington, VT 05401
(802) 847- 2554
